# Genetic variants in *PLCB4*/*PLCB1* as susceptibility loci for coronary artery aneurysm formation in Kawasaki disease in Han Chinese in Taiwan

**DOI:** 10.1038/srep14762

**Published:** 2015-10-05

**Authors:** Ying-Ju Lin, Jeng-Sheng Chang, Xiang Liu, Hsinyi Tsang, Wen-Kuei Chien, Jin-Hua Chen, Hsin-Yang Hsieh, Kai-Chung Hsueh, Yi-Tzone Shiao, Ju-Pi Li, Cheng-Wen Lin, Chih-Ho Lai, Jer-Yuarn Wu, Chien-Hsiun Chen, Jaung-Geng Lin, Ting-Hsu Lin, Chiu-Chu Liao, Shao-Mei Huang, Yu-Ching Lan, Tsung-Jung Ho, Wen-Miin Liang, Yi-Chun Yeh, Jung-Chun Lin, Fuu-Jen Tsai

**Affiliations:** 1 Genetic Center, Department of Medical Research, China Medical University Hospital, Taichung, Taiwan; 2 School of Chinese Medicine, China Medical University, Taichung, Taiwan; 3 Children’s Hospital of China Medical University, Taichung, Taiwan; 4 School of Medicine, China Medical University, Taichung, Taiwan; 5 National Institute of Allergy and Infectious Diseases, National Institutes of Health, Bethesda, Maryland, USA; 6 Biostatistics Center, College of Management, Taipei Medical University, Taipei, Taiwan; 7 School of Health Care Administration, College of Management, Taipei Medical University, Taipei, Taiwan; 8 Pediatric Emergency Division of Children’s Hospital, China Medical University, Taichung, Taiwan; 9 Kai-Chung Hsueh Clinics, Taichung, Taiwan; 10 Heart Center, China Medical University Hospital, Taichung, Taiwan; 11 Rheumatism Research Center, China Medical University Hospital, Taichung, Taiwan; 12 Department of Medical Laboratory Science and Biotechnology, China Medical University, Taichung, Taiwan; 13 Department of Microbiology and Immunology, Graduate Institute of Biomedical Sciences, Chang Gung University, Taoyuan, Taiwan; 14 Institute of Biomedical Sciences, Academia Sinica, Taipei, Taiwan; 15 Department of Health Risk Management, China Medical University, Taichung, Taiwan; 16 Graduate Institute of Biostatistics, School of Public Health, China Medical University, Taichung, Taiwan; 17 School of Medical Laboratory Science and Biotechnology, College of Medical Science and Technology, Taipei Medical University, Taipei, Taiwan; 18 Department of Biotechnology and Bioinformatics, Asia University, Taichung, Taiwan

## Abstract

Kawasaki disease (KD) is an acute, inflammatory, and self-limited vasculitis affecting infants and young children. Coronary artery aneurysm (CAA) formation is the major complication of KD and the leading cause of acquired cardiovascular disease among children. To identify susceptible loci that might predispose patients with KD to CAA formation, a genome-wide association screen was performed in a Taiwanese KD cohort. Patients with both KD and CAA had longer fever duration and delayed intravenous immunoglobulin treatment time. After adjusting for these factors, 100 susceptibility loci were identified. Four genes were identified from a single cluster of 35 using the Ingenuity Pathway Analysis (IPA) Knowledge Base. Silencing *KCNQ5*, *PLCB1*, *PLCB4*, and *PLCL1* inhibited the effect of lipopolysaccharide-induced endothelial cell inflammation with varying degrees of proinflammatory cytokine expression. *PLCB1* showed the most significant inhibition. Endothelial cell inflammation was also inhibited by using a phospholipase C (PLC) inhibitor. The single nucleotide polymorphism rs6140791 was identified between *PLCB4* and *PLCB1*. Plasma PLC levels were higher in patients with KD and CC+CG rs6140791genotypes, and these genotypes were more prevalent in patients with KD who also had CAA. Our results suggest that polymorphism of the *PLCB4/B1* genes might be involved in the CAA pathogenesis of KD.

Kawasaki disease (KD; MIM 611775) is an acute, inflammatory, and self-limited vasculitis predominantly affecting infants and young children^1^,^2^,^3^. Prolonged fever, polymorphous skin rash, and swollen glands, hands, and feet are also observed in these patients. Coronary artery aneurysm(CAA) is the major complication of KD and have made KD the leading cause of acquired cardiovascular complications among children in industrialized countries^4^. Much effort has been directed toward decreasing CAA formation in KD. Currently, the only effective evidence-based treatment is administration of aspirin and intravenous immunoglobulin (IVIG) during the acute stage of KD, which abrogates the inflammation and reduces coronary artery damage to less than 5%^5^. Although the etiology and pathogenesis of KD remain poorly understood, it is believed that an abnormal and sustained inflammatory stimulus leads to host immune dysregulation in genetically susceptible individuals. During the acute stage, the infiltration of T cells and macrophages and the activation of vascular endothelium cells (ECs) with increased serum proinflammatory cytokines lead to inflammation and damage, with small- and medium-sized vessels along with those of the coronary artery being predominantly affected^6^,^7^. The injured vascular tissues show subendothelial edema, vascular damage, gap formation, and fenestration of ECs, all of which contribute to the pathogenesis of this disorder^8^,^9^. Therefore, identification of predisposing genetic factors might greatly improve the understanding of this disease and the formation of CAA therein.

Several host genetic factors have been identified that contribute to KD susceptibility through the use of genome-wide screens^10^,^11^,^12^,^13^,^14^,^15^. Susceptibility loci related to immune activation, inflammation, apoptosis, and cardiovascular pathology have been reported in Caucasian children with KD^10^,^14^. In addition, predisposing loci related to immune activation, inflammation, T cell receptor signaling, regulation of proinflammatory cytokines, and antibody-mediated immune responses have also been described in Asian children with KD^12^,^13^,^15^,^16^. It has been further noted through population-based studies in Taiwan that children with KD tend to have higher risks of subsequent allergic susceptibilities including atopic dermatitis (AD), allergic rhinitis (AR), asthma, and urticaria after KD illness^17^,^18^,^19^,^20^. These KD predisposing loci might also contribute to determinants of allergic disease with distinct immune phenotypes.

Although the cause of KD remains unknown, many efforts have been made to decrease CAA formation in patients with KD by using aspirin and IVIG treatments. In addition, proposed candidate gene studies for CAA formation in KD have suggested the involvement of genetic factors including *MICB*, *PELI1*, *CASP3*, *CD40*, *MMP-3*, *MMP-12*, HLA-B associated transcript 2, 3, and 5, *ITPR3*, *HLA-E*, *HLA-G*, *ITPKC*, *IL-10*, and angiotensin I converting enzyme (*ACE*) genes^11^,^16^,^21^,^22^,^23^,^24^,^25^,^26^,^27^,^28^,^29^,^30^. These candidate genes are involved in the immune-regulatory responses and cardiovascular-related pathogenesis that contribute to the susceptibility to and/or formation of CAA in KD. To search for additional loci representing novel mechanisms that might predispose patients with KD to CAA formation, we performed a genome-wide screen to identify novel CAA susceptibility loci in a Han Chinese population in Taiwan, evaluated the relationships between clinical characteristics and aneurysm formation in patients and selected loci, and functionally characterized the associated genes to determine their potential as regulators of proinflammatory cytokine expression in vascular ECs.

## Results

### Summary data for the newly identified loci associated with CAA formation in Taiwanese Kawasaki disease

**Figure 1:**
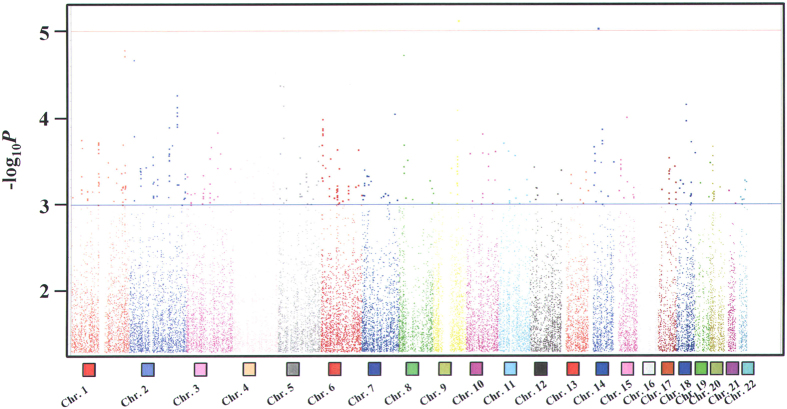
Genome-wide screening results. Manhattan plot for the SNPs on autosomal chromosomes, obtained using the chi-square test under a dominant model. The red and blue horizontal lines indicate the threshold of the genome-wide screen (*p* < 1 × 10^−5^) and the cutoff level for the top 203 SNPs used for the following functional studies (*p* < 0.005), respectively.

**Figure 2:**
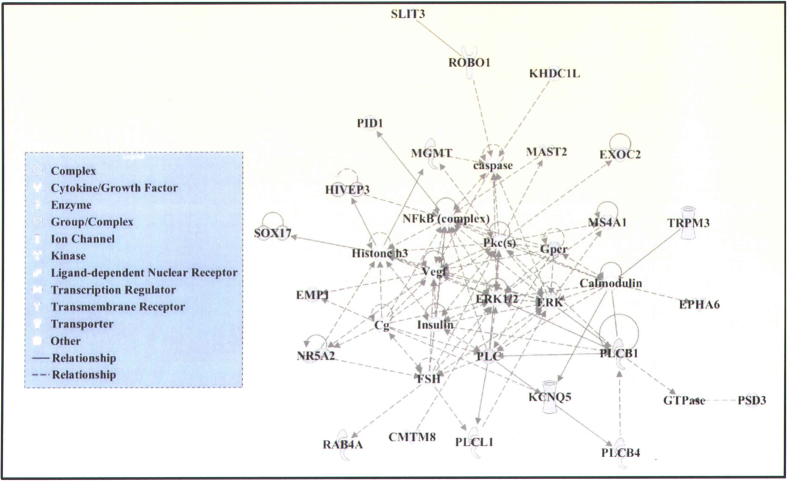
Putative gene network derived from Ingenuity Pathway Analysis (IPA) software. IPA network analysis identified a single cluster of 35 genes that includes 26 associated genes discovered in this study. The lines between genes represent known interactions (solid lines represent direct interactions; dashed lines represent indirect interactions). Each gene is displayed using various shapes that represent the functional class of the gene product, as indicated in the legend.

To identify the susceptibility gene loci associated with KD related CAA complications, we performed chi-square tests for allelic and genotypic comparisons under the dominant model in a Han Chinese population with KD residing in Taiwan. Stratification of patients for CAA according to either the right or the left coronary artery identified a dilation diameter ≥3 mm in children younger than 5 years of age, or ≥4 mm in older children^35^,^31^. As shown in Table S1, significant clinical factors associated with KD CAA formation were fever duration (*p* < 0.0001) and time of first IVIG treatment (days after the first day of fever) (*p* < 0.0001). After adjusting for these potential factors by using multivariate regression analyses, significant associations from genome-wide association tests were observed (Fig. 1). A total of 203 single nucleotide polymorphisms (SNPs) that reached *p* < 0.005 in the genome-wide screen of KD with CAA versus without CAA are shown in Table S2. From these, we selected the top 100 SNPs (that reached *p* < 0.003) for possible pathway mapping and further functional validation. We explored possible functional relationships between the 100 SNP-related genes by using the Ingenuity Pathway Analysis (IPA) Knowledge Base (Table 1). The IPA network analysis identified a single cluster of 35 genes that included 26 associated genes discovered in this study (Fig. 2). From among these we selected 4 genes, *KCNQ5*, *PLCB1*, *PLCB4*, and *PLCL1*, for further functional characterization according to the criteria that at least two SNPs were identified in the GWAS study from the same gene and that their *p* values reached *p* < 5 × 10^−4^ (Table 1).

### Effect of *KCNQ5*, *PLCB1*, *PLCB4*, and *PLCL1* knockdown on *IL-1*, *IL-6,* and *IL-8* proinflammatory cytokine mRNA expression

**Figure 3:**
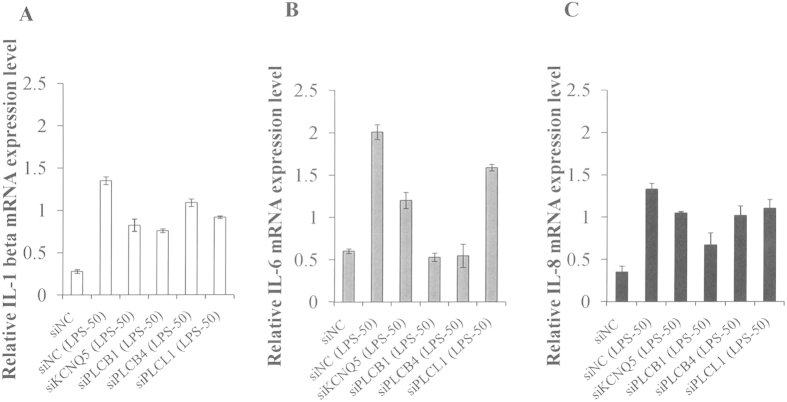
Effect of *KCNQ5*, *PLCB1*, *PLCB4*, and *PLCL1* down-regulation on *IL-1*, *IL-6*, and *IL-8* proinflammatory cytokine mRNA expression. HUVEC cells were transfected with siKCNQ5, siPLCB1, siPLCB4, and siPLCL1 or siNC for 24 h application of LPS for an additional 24 h. *IL-1beta* (**A**), *IL-6* (**B**), and *IL-8* (**C**) mRNA expression levels were quantified by RT-qPCR. Data represent means ± SD for three independent experiments.

EC injury and inflammation are some of the major characteristics of the development of KD^25^. When ECs were stimulated with pathogenic mediators such as lipopolysaccharide (LPS), the stimulated cells were shown to trigger inflammatory signals to increase permeability and leukocyte recruitment^32^. In this study, we examined 4 genes, *KCNQ5*, *PLCB1*, *PLCB4*, and *PLCL1*, for their effect on proinflammatory cytokine mRNA expression by silencing them using siRNA (Fig. 3 and Supplementary Fig. S1) in an LPS-induced endothelial cell inflammation model. Among them, *PLCB1* showed the most significant inhibition of *IL-1 beta*, *IL-6*, and *IL-8* expression. These results suggest that *PLCB1* might regulate endothelial cell inflammation via interference with proinflammatory cytokine expression. This is the first study to report that *PLCB1* is a regulator of vascular inflammation and thus its use might be beneficial for many inflammatory diseases associated with endothelial dysfunction.

### Plasma levels of phospholipase C (PLC) and the effect of a PLC inhibitor on *IL-1*, *IL-6*, and *IL-8* proinflammatory cytokine mRNA expression

**Figure 4:**
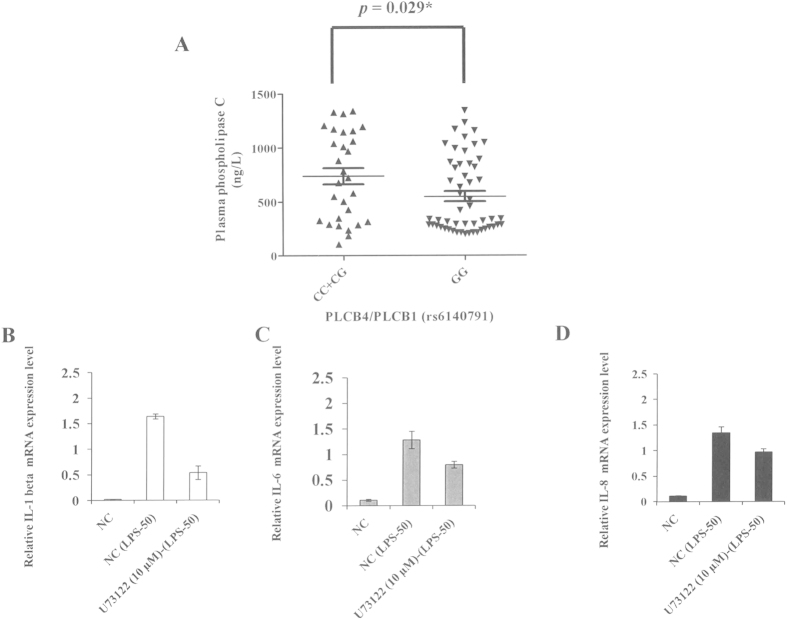
Plasma levels of phospholipase C (PLC) and the effect of a PLC inhibitor on *IL-1*, *IL-6*, and *IL-8* proinflammatory cytokine mRNA expression. (**A**) Detection of PLC in plasma from patients with KD. PLC concentrations in plasma samples from 96 patients with KD are shown in relation to genotypes. *P* values were determined by student’s t test. B) HUVEC cells were treated with PLC inhibitor for 24 h followed by treatment with LPS for another 24 h. *IL-1beta* (**B**), *IL-6* (**C**) and *IL-8* (**D**) mRNA expression levels were quantified by RT-qPCR. Data represent means ± SD for three independent experiments.

The molecular interactions between PLCB1, PLCB4, and PLC and their effect on proinflammatory cytokine production have been previously investigated^33^,^34^,^35^,^36^,^37^. Furthermore, as shown in Fig. 2, the PLCB1-PLC interaction might then regulate the NF-κB complex and affect the production of IL-1, IL-6, and IL-8 proinflammatory cytokines. Plasma PLC concentrations were therefore measured in 96 patients with KD by enzyme-linked immunosorbent assays (ELISAs). Phospholipase C levels in plasma samples from patients with KD were analyzed in relation to *PLCB4/B1* (rs6140791) genotypes (Fig. 4A). In patients with KD, those with CC+CG genotypes appeared to have significantly higher plasma levels of PLC than did those with the GG genotype (CC+CG: 738.7; GG: 549.7; *p* = 0.029). To investigate the effects of PLC inhibitor application on the IL-1, IL-6, and IL-8 proinflammatory cytokine production via the NF-κB complex, the PLC inhibitor (U73122) was used to treat ECs. LPS was then added to induce an EC inflammation model. As shown in Fig. 4B–D, U73122 mediated significant inhibition of *IL-1 beta*, *IL-6*, and *IL-8* expression. Thus, the PLC inhibitor U73122 might regulate EC inflammation via interference with proinflammatory cytokine expression.

### *PLCB4/B1* polymorphism and CAA severity

**Figure 5:**
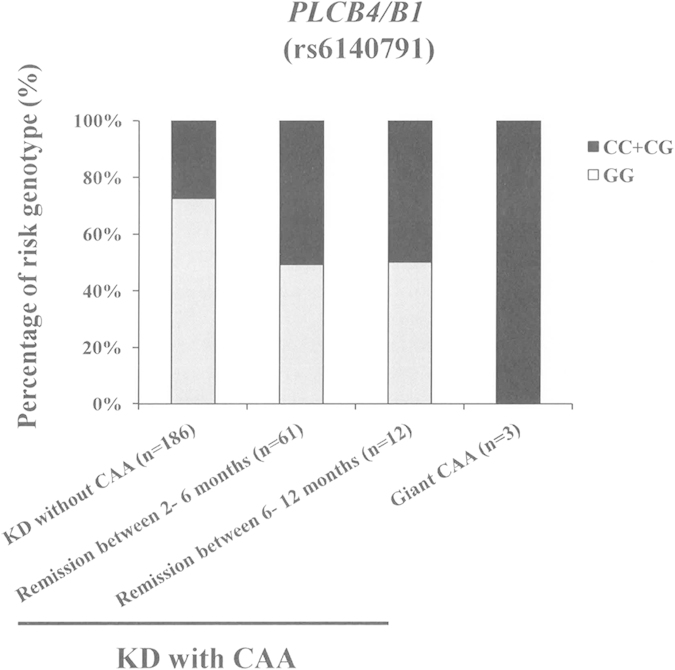
Distribution of the rs6140791 risk genotype frequency in the *PLCB4*/*B1* gene according to CAA severity. CAA was classified as an >3 or >4 mm increase in coronary artery diameter in children under or over 5 years of age during the first 2 months of KD diagnosis, respectively^31^,^55^. CAA severity grade: KD without CAA indicates patients with no CAA complications; KD with CAA (remission between 2–6 months) indicates patients with CAA, but who showed remission between 2–6 months after KD illness; KD with CAA (remission between 6–12 months) indicates patients with CAA, but who showed remission between 6–12 months after KD illness; KD with CAA (giant CAA) indicates patients with giant CAA (≥8 mm) or severe stenosis or occlusion. The genotypes of rs6140791 are shown according to CAA severity, and the number of patients in each category are indicated.

From our genome wide analysis, we identified one locus as being associated with CAA, the rs6140791 SNP located between the *PLCB4* and *PLCB1* genes on chromosome 20p12. As shown in Table 1, the frequencies of the CC+CG genotypes of this polymorphism were significantly higher in patients with KD with CAA than in those without CAA (OR = 2.861; 95% CI = 1.597–5.125; *p* = 4.08 × 10^−4^). The distributions of the CC+CG genotypes of this SNP were further analyzed according to CAA severity (Fig. 5). As compared with the KD without CAA group, the risk genotypes (CC+CG) of the significantly associated SNP rs6140791 were found to be enriched in the patients with KD with CAA remission between 2–12 months and also in those with giant CAA.

## Discussion

Genome-wide association analysis has enabled the discovery of novel genetic loci that contribute to KD susceptibility^10^,^11^,^12^,^13^,^14^,^15^. More recently, genetic susceptibility for the development of CAA formation has also been investigated using candidate gene analyses^11^,^16^,^21^,^22^,^23^,^24^,^25^,^26^,^27^,^28^,^29^,^30^. These studies have provided us with a preliminary genetic architecture of KD as well as of CAA formation therein. In the present work, we identified susceptibility loci that might contribute to CAA formation in Taiwanese patients with KD and identified one locus (rs6140791), located between the *PLCB4* and *PLCB1* genes, which showed likely contribution through functional analysis. Individuals carrying the CC+CG genotypes of this polymorphism had higher levels of plasma PLC; furthermore, we also observed a greater number of individuals with the CC+CG genotypes in the group of KD patients with CAA than in those without CAA. In addition, we showed that the effect of LPS-induced EC inflammation was highly significantly inhibited by application of siPLCB1 or PLC inhibitor, suggesting that the *PLCB4/B1* genes might play a role in the CAA pathogenesis of KD.

Genome-wide association studies have been applied in children with KD to identify CAA-associated loci^38^,^39^. Lin and colleagues performed a genome-wide association study using patients with KD with extremely large aneurysms (diameter > 8 mm; n = 64) and those without CAA (n = 70)^38^. Their work led to the discovery that the genetic variant rs2833195 in the intron of the *TIAM1* gene was associated with the development and severity of CAA in KD. Their results implied that the TIAM1 protein might be required for chemokine-induced T-cell migration and the lymphocyte infiltration into the arterial wall during the acute stage of KD. In addition, Kim and colleagues found that the genetic variant rs17136627 of the *KCNN2* gene was associated with CAA development in KD also by using patients with KD with only very large aneurysms (diameter > 5 mm; n = 17) and those without CAA (n = 123) for their KD CAA genome-wide screen^39^. They suggested that the as the *KCNN2* gene is highly expressed in arterial myocytes it might be associated with heart disease such as arrhythmias. However, the biological role of the *KCNN2* gene in KD pathogenesis remains to be elucidated. Because of limited sample numbers and lack of functional analyses, the significance of these newly identified CAA-associated loci remain inconclusive. In the present work, we analyzed genome-wide screen data generated from a larger sample size. We also adjusted the potential genetic factors for clinical factors such as fever duration and the time of first IVIG use as these factors have been previously reported to be associated with CAA formation in KD^40^,^41^,^42^. From this analysis, we identified CAA susceptibility loci in a Taiwanese cohort of patients with KD; we further characterized these novel genes, and identified *PLCB4* and *PLCB1* as important regulators of proinflammatory cytokine expression in vascular ECs.

Patients with KD tend to have abnormal serum lipid profiles and are associated with important abnormalities in lipid metabolism^43^,^44^,^45^,^46^. Serum lipid profiles such as triglycerides, total cholesterol, high-density lipoprotein-cholesterol (HDL-C), and low-density lipoprotein cholesterol (LDL-C) levels have been used as predictors of atherosclerosis, and have also been associated with cardiovascular diseases. As previously reported, patients with KD and coronary abnormalities have decreased HDL-C levels compared with those without such aneurysms^45^,^46^. Furthermore, another study reported that the levels of total cholesterol, LDL-C, and apolipoprotein B were higher in patients with KD and coronary abnormalities than in those without such aneurysms^44^. In the present work, we found 100 susceptible loci that were associated with KD CAA. Among them, we observed that a single cluster of 35 genes was associated with the top networks, i.e., embryonic development, nervous system development and function, organ development, and lipid metabolism by using the Ingenuity Pathway Analysis (IPA) Knowledge Base (Table S3). Our KD CAA genome-wide findings suggest that these potential CAA-associated gene variants might affect not only the severity of vasculitis during the acute stage of KD, but also might be related to lipid metabolism after KD illness. Children with these potential gene susceptibility variants might have high lipid profiles and arterial stiffness subsequent to KD, indicating an increased risk for cardiovascular disease.

In the present work, we chose 4 lipid metabolism associated genes, *KCNQ5*, *PLCB1*, *PLCB4*, and *PLCL1*, from among the highlighted gene cluster for further functional investigation. We used an LPS-induced EC inflammation model and knockdown of *KCNQ5*, *PLCB1*, *PLCB4*, and *PLCL1* by siRNA and found that these genes might regulate EC inflammation via interference with proinflammatory cytokine expression. Of these, the *PLCB1* gene showed the most significant inhibition. Consistent with this, our results also showed that EC inflammation was inhibited by using a PLC inhibitor as well. PLCB1 is expressed in cardiomyocytes, and overexpression of this protein causes cardiomyocyte hypertrophy. For synovial fibroblasts, macrophages, dendritic cells, and ECs, inflammation induced by thrombin, bradykinin, LPS, porphyromonas gingivalis, or advanced glycation end products has been shown to be affected by PLC inhibitors, suggesting that PLC might also be involved in NF-κB activation and proinflammatory cytokine release in inflammatory diseases^33^,^34^,^35^,^36^,^37^. The SNP rs6140791 is located between the *PLCB4* and *PLCB1* genes. KD patients carrying the CC+CG genotypes of this polymorphism appeared to have higher plasma PLC levels than did those with the GG genotype. In addition, the KD with CAA group tended to have more individuals with CC+CG genotypes than did the group without CAA. The CC+CG genotypes were further enriched in the patients with KD who exhibited CAA remission between 2–12 months and also in the patients with KD and giant CAA, suggesting that individuals carrying CC+CG genotypes might have higher expression levels of PLC. In turn, people with higher levels of PLC might have more severe inflammations and more potential to develop CAA in KD. This is the first study to report that *PLCB1* is a regulator of vascular inflammation and to suggest that its use might therefore be beneficial for the management of inflammatory diseases associated with endothelial dysfunction.

KD is the leading cause of acquired cardiovascular diseases among children with a possible underlying pathology of immune-mediated vasculitis^1^,^47^. Endothelial cell injury and inflammation play important roles in the development of KD as well as in CAA formation^25^. When ECs were induced by pathogenic mediators including LPS, the cells were shown to activate inflammatory signals to increase permeability and leukocyte recruitment^32^. In this study, functional investigation suggested that RNA silencing of *PLC* genes or pharmacologic inhibition of PLC downregulated EC inflammation. The PLC gene, *PLCB1*, consists of 36 exons and localizes to 20p12. *PLCB1* codes for the PLC beta 1 protein, which represents one of the four PLC beta isoforms (PLC beta 1–4), has two transcript variants comprised of 1216 and 1173 residues that produce different proteins. PLC beta plays important roles in cardiomyocytes, synovial fibroblasts, macrophages, dendritic cells, and endothelial cells^33^,^34^,^35^,^36^,^37^,^48^,^49^. Furthermore, genetic variants in *PLCB4/PLC1* have been significantly associated with several phenotypic traits including levels of apolipoprotein B, cholesterol and HDL, along with body weight, body mass index and stroke (Table S4). For cardiomyocytes, PLC beta is the immediate downstream target of Gαq, a heterotrimeric G protein that regulates its activation, and hydrolyzes the plasma membrane phosphatidylinositol 4,5-bisphosphate (PIP2) protein to produce the second messengers inositol 1,4,5-triphosphate (IP3; a regulator of the intracellular calcium response) and diacylglycerol (DAG, an activator of protein kinase C subtypes)^50^. Studies have reported that PLC beta might transmit the cardiac hypertrophy signal initiated by Gαq^48^,^51^. Our findings are also in agreement with these studies. Our results demonstrated that the effect of LPS-induced EC inflammation was inhibited with the most significant inhibition by using siPLCB1 or a PLC inhibitor, suggesting that PLCB1 might be involved in EC inflammation, a possibility that has important implications for understanding the pathogenesis of immune-mediated vascular diseases.

In conclusion, we have identified a gene locus that is associated with a genetic predisposition to the development of CAA in KD in Taiwanese children of Han Chinese ethnic background. Functional analysis also supports the possibility that *PLCB1* might predispose patients with KD to CAA formation.

## Methods

### Study population

All experiments were performed in accordance with the relevant guidelines and regulations. This study was approved by the Human Studies Committee of China Medical University Hospital. Written informed consent was obtained from the participants or their parent or legal guardian. Two hundred and sixty-two unrelated patients fulfilling the KD diagnostic criteria were identified and enrolled from the Children’s Hospital of China Medical University in Taichung, Taiwan^25^,^42^,^52^,^53^,^54^. There were 76 patients with KD with CAA complications and 186 patients with KD without CAA complications, with an average age at onset of 1.86 ± 1.78 and 1.70 ± 1.51 years old, respectively (Table S1). All patients were diagnosed according to KD criteria^55^,^56^, including fever lasting 5 days or more and at least 4 of the following symptoms: (1) changes in extremities (e.g., erythema, edema, or desquamation), (2) bilateral conjunctivitis, (3) polymorphous rash, (4) cervical lymphadenopathy, and (5) changes in the lips or oral cavity (e.g. pharyngeal erythema, dry/fissured or swollen lips, or “strawberry tongue”). All patients with KD received IVIG treatment and had regular echocardiographic examinations during the 1-year follow-up period. Echocardiographic examinations were completed during the acute stage, at 2 and 6 months after onset, and once per year thereafter. CAA was identified when either the right or the left coronary artery showed a dilated diameter ≥3 mm in children younger than 5 years of age, or ≥4 mm in older children^31^,^55^, regardless of whether the CAA went into remission between 2–12 months after KD illness. Only Han Chinese individuals, who account for 98% of Taiwanese residents, were recruited. The ethnic background was assigned based on the results of self-reported questionnaires. The characteristics and clinical profiles of patients with KD enrolled in the study are summarized in Table S1. Statistically significant differences were observed for fever duration (*p* < 0.0001), first time of IVIG treatment (days after the first day of fever) (*p* < 0.0001), and efficacy of the first IVIG treatment (*p* = 0.002). No significant differences were observed in our study for laboratory test results obtained at the acute stage within 24 h before IVIG treatment and for KD status within 3–7 days after IVIG treatment.

### Genotyping and quality control

Genomic DNA was extracted from patient blood according to standard protocols by using the Qiagen Genomic DNA Isolation Kit (Qiagen, Taichung, Taiwan). For the genome-wide screen, each sample was genotyped by the National Genotyping Center at Academia Sinica (Taipei, Taiwan) using the Affymetrix Genome-Wide Human SNP Array 6.0, which contains a total of 906,600 SNPs, according to the manufacturer’s procedure. Genotype data were quality controlled and SNPs were excluded for further analysis if (1) only one allele appeared in patients; (2) the total call rate was less than 95%; (3) the total minor allele frequency was less than 5%; or (4) the SNP significantly departed from Hardy-Weinberg equilibrium proportions (*p* < 0.05).

### Statistical analysis

Data were expressed as indicated for continuous variables (Table S1). Chi-squared tests were used to identify differences in categorical variables, and ORs and 95% CIs were calculated for the factors under consideration. Genotypes were obtained by direct counting followed by forward stepwise multivariate regression analyses for adjusting clinical factors (fever duration and first time of IVIG use) (Table 1). All statistical analyses were performed using SPSS v12.0 for Windows (IBM, Armonk, NY, USA).

### Cells

HUVECs (BCRC Number: H-UV001) were grown in 90% GIBCO medium 199 (Life Technologies) with 25 U/mL heparin (Sigma, St. Louis, MO, USA), 30 μg/mL endothelial cell growth supplement (Millipore, Billerica, MA, USA) adjusted to contain 1.5 g/L sodium bicarbonate + 10% fetal bovine serum and 100 U/mL penicillin/streptomycin.

### Short Interfering RNA

siRNA targeting transcripts for *KCNQ5*, *PLCB1*, *PLCB4*, and *PLCL1* (5′–3′; siKCNQ5: duplex 1, GGGCAAAUCACAUCAGAUA; duplex 2, CAACACAGGUGCCAAUUAG; duplex 3, GACAUGUUGUGUAGAAUUA; duplex 4, GGGAGGCACUUGGAAAUUA; siPLCB1: duplex 1, GAAGAUAACAGAAGCUAAA; duplex 2, GCAAUUGGCUGCUUUGACA; duplex 3, GAUGAUGACUCAACUAUUG; duplex 4, CAACAGAAAUCGUUUGUGA; siPLCB4: duplex 1, GUAAUUGUCUCGAAAUGAA; duplex 2, GAGAAUAGCUGUGUAUGAU; duplex 3, CAAGAAAGGUAUUGAACUU; duplex 4, CCACUAAUAUCCAUCCAUA; siPLCL1: duplex 1, GCACAGAAGCGCAGUCUUU; duplex 2, GGUAAUGGCUCAACAGAUG; duplex 3, GAAGAAAGUUCGGGAAUAU; duplex 4, GUAGGGAGCUCUCUGAUUU) were purchased from Invitrogen (Carlsbad, CA, USA), as were the non-targeting siRNA scrambled controls (siNC: duplex 1, AUGAACGUGAAUUGCUCAA; duplex 2, UAAGGCUAUGAAGAGAUAC; duplex 3, AUGUAUUGGCCUGUAUUAG; duplex 4, UAGCGACUAAACACAUCAA).

### Endothelial cell inflammation assay

To measure endothelial cell inflammation, HUVECs were aliquoted in 6-well plates. Cells were transfected with the desired siRNAs or siNCs as controls using Lipofectamine 2000 (Invitrogen). The transfected cells were then treated with 50 μg/mL LPS for another 24 h. Cellular RNA extraction and real-time reverse transcription (RT)-PCR analyses were performed as described below.

### Real-time RT-PCR

Cellular RNAs were isolated using a QIAamp® RNA Mini Kit according to the manufacturer’s instructions (Qiagen, Valencia, CA, USA). RNAs were eluted in 60 μL buffer, and real-time TaqMan RT-PCR assays were used to determine the siRNAs knock-down efficiency. The primers used for quantitative PCR (qPCR) amplification were as follows: *KCNQ5*, forward: 5′- AGGGGAAGCATCAAGCAGTA -3′ and reverse: 5′- CGCACTCGCTCCTTAAAACT -3′; *PLCB1*, forward: 5′- GTGGGAGACACGCCAAAG -3′ and reverse: 5′- GGCCCATACACCACTGTGA -3′; *PLCB4*, forward: 5′- CGGGAAGTCTTCGGTAGAAA -3′ and reverse: 5′- CCCAGCAGTCAAGTTCAACA -3′; *PLCL1*, forward: 5′- TCACTTGTGATGAAAGACAGCTT -3′ and reverse: 5′- GAGAAACCGGCTCTCTTGAA -3′; *IL-1 beta*, forward: 5′-TACCTGTCCTGCGTGTTGAA-3′ and reverse: 5′-TCTTTGGGTAATTTTTGGGATCT -3′; *IL-6*, forward: 5′- CAGGAGCCCAGCTATGAACT -3′ and reverse: 5′- GAAGGCAGCAGGCAACAC -3′; *IL-8*, forward: 5′- GAGCACTCCATAAGGCACAAA -3′ and reverse: 5′- ATGGTTCCTTCCGGTGGT -3′. Reverse transcription was performed in a 10 μL reaction mixture consisting of 2 μL RNA template, 1 μL RT primer mix, 1 μL dNTP mix (10 mm each), and 6 μL RNA/DNAse-free water at 65 °C for 5 min. Next, a reaction mixture of 4 μL 5 × MMLV buffer, 0.8 μL MMLV enzyme, and 5.2 μL RNA/DNAse-free water was added to each RNA sample. Reverse transcription reactions were performed at 42 °C for 60 min. cDNA was amplified by PCR in a 20 μL reaction mixture containing 5 μL cDNA, 10 μL, 2 × Mastermix, 1 μL primer/probe mix, and 4 μL RNA/DNAse-free water. Real-time TaqMan RT-PCR conditions were 95 °C for 10 min, and 50 cycles of 95 °C for 10 s, and 60 °C for 60 s. RNA levels were detected using a 7900HT Fast Real-Time PCR System (Life Technologies, Carlsbad, CA, USA).

### *In vivo* and in *vitro* PLC analysis

Serum PLC levels in patients with KD were assessed using a Human Phospholipase C (PLC) Enzyme-linked Immunosorbent Assay (ELISA) kit (Cat. No: E0788Hu) obtained from Shanghai Crystal day Biotech Co., Ltd. (Shanghai, China) according to manufacturer instruction (http://www.bt-laboratory.com/admin/upload/201212139414181958.pdf). For determination of the *in vitro* effects of the PLC inhibitor U73122 (Cat. No: U 6756, Sigma), HUVEC cells were treated with10 μM for 24 h at 37 °C followed by 50 μg/mL LPS for another 24 h., followed by qRT-PCR analysis.

## Additional Information

**How to cite this article**: Lin, Y.-J. *et al.* Genetic variants in *PLCB4/PLCB1* as susceptibility loci for coronary artery aneurysm formation in Kawasaki disease in Han Chinese in Taiwan. *Sci. Rep.* **5**, 14762; doi: 10.1038/srep14762 (2015).

## Supplementary Material

Supplementary Information

## Figures and Tables

**Table 1:** Association results for the 100 SNPs in GWAS analysis of KD with CAA and without CAA.

**SNP**	**Chr.**	**Cytoband**	**Position**^a^	**Gene**^b^	**Gene Relationship**	**Genotype**	**P**	**OR**
rs11210499	1	p34.2	41624031	HIVEP3	intron	C/T	4.65E-04	0.3494
rs11264793	1	q23.1	157677735	FCRL4/FCRL3	upstream/downstream/UTR-3/intron	A/T	3.21E-04	2.946
rs7549100	1	q23.1	157688051	FCRL3	intron	A/G	4.74E-04	2.826
rs10801121	1	q31.2	192509743	RGS1	upstream/downstream	A/G	3.80E-04	2.972
rs7537542	1	q32.1	199271032	LOC100131234/NR5A2	upstream/intron	A/G	2.91E-04	0.3178
rs12095873	1	q32.1	202818914	LOC641515/KDM5B-AS1	upstream/downstream	C/T	0.001924	2.649
rs6693436	1	q41	219112207	LOC643723	intron	C/T	0.001407	3.045
rs12119303	1	q41	219141207	LOC643723	intron	G/T	9.90E-04	3.193
rs4846532	1	q41	219156014	LOC643723	intron	A/G	9.90E-04	3.193
rs6541205	1	q41	219160887	LOC643723	intron	C/T	9.90E-04	3.193
rs10746384	1	q41	219202204	LYPLAL1	intron	A/G	6.19E-04	3.373
rs1568804	1	q41	219217862	RNU5F-1/LYPLAL1	upstream/downstream	A/G	9.11E-04	3.216
rs6704354	1	q41	219220546	RNU5F-1/LYPLAL1	upstream/downstream	C/T	9.11E-04	3.216
rs6670457	1	q41	219229084	RNU5F-1/LYPLAL1	upstream/downstream	A/G	7.16E-04	3.335
rs11118243	1	q41	219233066	RNU5F-1/LYPLAL1	upstream/downstream	C/T	7.16E-04	3.335
rs16848112	1	q42.13	227747048	SNAP47	intron	C/T	1.64E-05	4.075
rs6674275	1	q42.13	227748758	SNAP47	intron	C/T	1.64E-05	4.075
rs12064596	1	q42.13	227756257	SNAP47	intron	A/G	1.91E-05	4.033
rs12064154	1	q42.13	229146328	RAB4A/RHOU	upstream/downstream	C/T	5.98E-04	2.76
rs2345493	2	p24.2	18110934	KCNS3/RDH14	downstream/intron	G/T	2.12E-05	4.971
rs2345496	2	p24.2	18135374	KCNS3/RDH14	downstream/intron	A/G	1.60E-04	4.047
rs10202102	2	p13.3	68784021	ARHGAP25	intron	C/T	3.65E-04	3.656
rs10174913	2	q14.1	116017590	DDX18/DPP10	upstream/downstream	A/C	5.01E-04	3.945
rs16831039	2	q33.1	199144083	PLCL1/SATB2	downstream	C/T	8.51E-05	3.31
rs1376584	2	q33.1	199144170	PLCL1/SATB2	downstream	C/T	8.51E-05	3.31
rs1901321	2	q33.1	199148856	PLCL1/SATB2	downstream	A/G	5.42E-05	3.472
rs921465	2	q33.1	199150704	PLCL1/SATB2	downstream	A/C	7.39E-05	3.391
rs1868913	2	q33.1	199162581	PLCL1/SATB2	downstream	A/T	8.51E-05	3.31
rs1901323	2	q33.1	199162766	PLCL1/SATB2	downstream/upstream	A/T	8.51E-05	3.31
rs10931853	2	q33.1	199163235	PLCL1/SATB2	downstream/upstream	C/T	8.51E-05	3.31
rs6730991	2	q33.1	199165025	PLCL1/SATB2	downstream/upstream	A/G	8.51E-05	3.31
rs16831114	2	q33.1	199166663	PLCL1/SATB2	downstream/upstream	C/T	9.34E-05	3.288
rs1584661	2	q33.1	199170501	PLCL1/SATB2	downstream/upstream	C/T	8.51E-05	3.31
rs6742079	2	q33.1	199173285	PLCL1/SATB2	downstream/upstream	A/G	8.51E-05	3.31
rs6717968	2	q33.1	199193218	PLCL1/SATB2	downstream	C/T	8.51E-05	3.31
rs10804088	2	q33.1	199203328	PLCL1/SATB2	downstream	A/G	8.51E-05	3.31
rs1376591	2	q33.1	199203436	PLCL1/SATB2	downstream	C/T	8.51E-05	3.31
rs4338918	2	q33.1	199208128	PLCL1/SATB2	downstream	A/T	8.51E-05	3.31
rs6796318	3	p22.3	32260293	CMTM8	intron	A/G	7.32E-04	3.415
rs4132830	3	p22.3	32273890	CMTM8	intron	C/T	0.002041	3.058
rs3821595	3	p12.3	78619433	ROBO1	intron	A/T	0.001671	0.3807
rs6807510	3	p12.3	78630434	ROBO1	intron	C/T	0.001684	0.3808
rs6793657	3	q21.3	127185449	PLXNA1/TPRA1/C3orf56	downstream/upstream	C/T	3.73E-04	0.2914
rs13072025	3	q21.3	127199552	PLXNA1/TPRA1/C3orf56	downstream	A/T	7.91E-04	0.3694
rs11923216	3	q21.3	127205138	PLXNA1/TPRA1	downstream	C/T	7.91E-04	0.3694
rs2411265	4	q12	52777311	ERVMER34-1/LOC152578	upstream	A/G	0.00185	2.517
rs17613967	4	q12	52779752	ERVMER34-1/LOC152578	upstream	G/T	0.001459	2.571
rs10028567	4	q12	52791409	LOC152578	intron	C/T	0.001459	2.571
rs4862161	4	q35.1	183349528	CLDN24/CDKN2AIP	upstream/downstream	C/T	4.35E-04	0.2932
rs17608672	5	p15.33	2645450	IRX4/IRX2	upstream/downstream	C/T	0.001117	3.916
rs9313144	5	p15.32	6132052	KIAA0947/FLJ33360	downstream/upstream	C/T	4.19E-05	0.2777
rs12514641	5	q23.1	120075328	PRR16/FAM170A	upstream/downstream	C/T	9.74E-04	0.3764
rs10045387	5	q23.1	120076291	PRR16/FAM170A	upstream/downstream/UTR-3/intron	A/G	6.86E-04	0.3629
rs356486	5	q31.2	139705517	PSD2/CXXC5	upstream/downstream/intron	C/G	0.002847	2.624
rs17668965	5	q35.1	169590225	CCDC99	intron	A/G	2.09E-04	3.032
rs2317217	6	p25.3	796483	EXOC2/LOC285768	upstream/downstream	A/C	2.05E-04	0.3254
rs7757332	6	p25.3	800495	EXOC2/LOC285768	upstream/downstream	C/T	0.001312	0.3595
rs688176	6	p25.2	4067874	PRPF4B/FAM217A	downstream/intron	C/T	1.51E-04	0.3233
rs593291	6	p25.2	4068481	FAM217A	UTR-3/intron/exon	A/C	1.56E-04	0.3187
rs595413	6	p25.2	4068931	FAM217A	missense/intron/cds/exon	C/T	1.32E-04	0.3189
rs2783063	6	p25.2	4080643	C6orf201/FAM217A	intron	G/T	5.27E-04	0.3568
rs11755877	6	p25.2	4081942	C6orf201/FAM217A /	intron	C/G	3.50E-04	0.347
rs101418	6	p25.2	4083037	C6orf201/FAM217A	intron	C/T	1.38E-04	0.3213
rs662834	6	p25.2	4083154	C6orf201/FAM217A	intron	C/G	3.50E-04	0.347
rs634114	6	p25.2	4086685	C6orf201/FAM217A	intron	C/G	1.38E-04	0.3213
rs707991	6	p25.2	4097677	C6orf201	intron	A/G	1.04E-04	0.3145
rs16896290	6	q12	65118594	EYS	intron	G/T	8.21E-04	3.272
rs841531	6	q12	65196436	EYS	intron	C/T	6.85E-04	3.196
rs10485313	6	q12	65210760	EYS	intron	A/G	6.03E-04	3.241
rs539248	6	q12	65218659	EYS	intron	A/T	6.57E-04	3.291
rs4142063	6	q12	65259036	EYS	intron	G/T	2.30E-04	3.609
rs6915695	6	q12	65269380	EYS	intron	A/T	2.30E-04	3.609
rs4991400	6	q13	73210711	KCNQ5/KHDC1L	downstream/intron	A/T	3.83E-04	0.3496
rs6453655	6	q13	73210829	KCNQ5/KHDC1L	downstream/intron	A/T	3.83E-04	0.3496
rs41420446	7	p15.1	28177760	JAZF1	intron	A/G	4.70E-04	0.2814
rs1868651	7	p14.1	37601817	ELMO1/GPR141	upstream	G/T	5.35E-04	0.3538
rs7795852	7	q34	140300704	LOC100134229/SLC37A3	downstream/intron	A/G	8.94E-05	3.768
rs6993670	8	p22	18871430	PSD3	intron	A/G	1.88E-05	4.184
rs10119687	9	q22.33	97710546	XPA/FOXE1	upstream/downstream	A/G	8.14E-05	3.555
rs7849782	9	q31.1	101664981	GRIN3A	intron	C/G	7.54E-06	0.2482
rs912745	10	q26.11	117738648	EMX2/RAB11FIP2	downstream	C/T	2.43E-04	4.945
rs217756	11	p15.1	16784612	C11orf58/PLEKHA7	downstream/intron	G/T	9.84E-05	4.557
rs520289	11	q23.3	117479421	DSCAML1	intron	A/G	5.12E-04	0.345
rs926150	12	p11.22	28087892	PTHLH/CCDC91	upstream	C/G	7.79E-04	0.3701
rs470393	12	q24.33	128972744	GLT1D1	intron	C/T	4.00E-04	3.818
rs12590437	14	q21.1	37977886	FOXA1/SSTR1/LOC100652860	upstream/intron	A/T	9.31E-06	4.009
rs10144855	14	q22.2	54745523	SAMD4A	intron	C/G	1.34E-04	3.159
rs17774131	16	q22.1	69469883	CYB5B/MIR1538/NFAT5	downstream/upstream	C/G	0.001114	2.674
rs12598083	16	q24.1	86397391	LOC732275/FOXF1-AS1	upstream/downstream	C/G	2.90E-05	3.615
rs11082212	18	q12.3	40803209	LOC647946/KC6 /	upstream/downstream	A/G	6.96E-05	4.123
rs1431301	18	q12.3	40818374	LOC647946/KC6 /	upstream/downstream	A/G	6.96E-05	4.123
rs2313647	18	q12.3	40819127	LOC647946/KC6 /	upstream/downstream	C/T	6.96E-05	4.123
rs17756653	18	q12.3	40820189	LOC647946/KC6 /	upstream/downstream	C/T	6.96E-05	4.123
rs323585	18	q12.3	40865874	LOC647946/KC6 /	upstream/downstream	A/G	1.08E-04	3.906
rs9951264	18	q12.3	44457712	SYT4/SETBP1	upstream/downstream	G/T	0.00149	0.3937
rs1030583	18	q21.33	62068449	PIGN	intron	C/G	1.88E-04	3.526
rs11659253	18	q23	77895725	SALL3/GALR1	upstream/downstream	C/T	2.52E-04	3.343
rs6140791	20	p12.3	8906575	PLCB4/PLCB1	upstream/downstream/intron	C/G	4.08E-04	2.861
rs4299396	20	p12.3	8934667	PLCB4/PLCB1	upstream/downstream/intron	A/T	2.12E-04	3.057
rs16995415	20	p12.3	8935154	PLCB4/PLCB1	upstream/downstream/intron	A/G	2.72E-04	2.962

Association results are ordered by the chromosome, cytoband, and position.

SNP, single nucleotide polymorphism; CAA, KD patients with coronary artery aneurysm; OR, odds ratio.

^a^Chromosome positions are based on NCBI GRCh38 version.

^b^Defined as the gene containing the SNP or the closest genes (within 100 kb up- and downstream) to the SNP.
