# Supplementary material for: Genetic variants in PLCB4/PLCB1 as susceptibility loci for coronary artery aneurysm formation in Kawasaki disease in Han Chinese in Taiwan
Source: Sci Rep. 2015 Oct 5;5:14762. doi: 10.1038/srep14762 (PMC4593004; doi:10.1038/srep14762)
Supplement: Supplementary Information [file srep14762-s1.pdf]

Genetic variants in *PLCB4*/*PLCB1* as susceptibility loci for coronary artery aneurysm formation in Kawasaki disease in Han Chinese in Taiwan

Ying-Ju Lin[1,2], Jeng-Sheng Chang[3,4], Xiang Liu[5], Hsinyi Tsang[5], Wen-Kuei Chien[6,7], Jin-Hua Chen[6,7], Hsin-Yang Hsieh[3,8], Kai-Chung Hsueh[9], Yi-Tzone Shiao[6], Ju-Pi Li[1,10] Cheng-Wen Lin[11], Chih-Ho Lai[12], Jer-Yuarn Wu[2,13], Chien-Hsiun Chen[2,13], Jaung-Geng Lin[2], Ting-Hsu Lin[1], Chiu-Chu Liao[1], Shao-Mei Huang[1], Yu-Ching Lan[14], Tsung-Jung Ho[2], Wen-Miin Liang[6], Yi-Chun Yeh[6] , Jung-Chun Lin[15] & Fuu-Jen Tsai[1,2,16,]*

[1]Genetic Center, Department of Medical Research, China Medical University Hospital, Taichung, Taiwan. [2]School of Chinese Medicine, China Medical University, Taichung, Taiwan. [3]Children's Hospital of China Medical University, Taichung, Taiwan. [4]School of Medicine, China Medical University, Taichung, Taiwan. [5]National Institute of Allergy and Infectious Diseases, National Institutes of Health, Bethesda, Maryland, USA. [6]Biostatistics Center and School of Public Health, China Medical University, Taichung, Taiwan. [7]Biostatistics Center and School of Public Health, Taipei Medical University, Taipei, Taiwan. [8] Pediatric Emergency Division of Children's Hospital, China Medical University, Taichung, Taiwan. [9] Kai-Chung Hsueh Clinics, Taichung, Taiwan. [10]Rheumatism Research Center, China Medical University Hospital, Taichung, Taiwan. [11]Department of Medical Laboratory Science and Biotechnology, China Medical University, Taichung, Taiwan. [12]Department of Microbiology, School of Medicine, China Medical University, Taichung, Taiwan. [13]Institute of Biomedical Sciences, Academia Sinica, Taipei, Taiwan. [14]Department of Health Risk Management, China Medical University, Taichung, Taiwan. [15]School of Medical Laboratory Science and Biotechnology, College of Medical Science and Technology, Taipei Medical University, Taipei,

Taiwan. [16]Department of Biotechnology and Bioinformatics, Asia University, Taichung, Taiwan.

*Correspondence and requests for materials should be addressed to Fuu-Jen Tsai, MD, PhD, Genetic Center, Department of Medical Research, China Medical University Hospital, Taichung, Taiwan. No. 2, Yuh Der Road, Taichung, Taiwan. Tel.: +886 4-22052121 ext. 2041; Fax: +886 4-22033295.

E-mail: d0704@mail.cmuh.org.tw

Table S1. Clinical characterististics of CAA-positive and CAA-negative individuals with Kawasaki disease in Taiwan

	Kawasaki disease		*p* value
	CAA-	CAA+	
**Number**	186	76	
**Age at Kawasaki disease diagnosis (years)[b]**	1.70±1.51	1.86±1.78	0.389
**Gender[a]**			
**Male (Number (%))**	120 (64.52%)	54 (71.05%)	0.387
**Female (Number (%))**	66 (35.48%)	22 (28.95%)	
**Fever duration (mean±SD, days)[b]**	7.48±2.67	10.61±4.90	***<0.0001***
**1st IVIG used time (mean±SD, days after the first date with fever)[b]**	6.31±2.24	8.14±4.10	***<0.0001***
**Laboratory test (Acute KD within 24 h before IVIG treatment)**			
**WBC (median (Q1-Q3), counts /μL)[c]**	14510 (10920-20800)	13600 (11110-17040)	0.243
**Hb (mean±SD, gm/dL)[b]**	11.11±1.34	11.17±1.28	0.747
**Hct (mean±SD, %)[b]**	32.64±4.00	33.09±3.35	0.415
**PLT (median (Q1-Q3), counts/μL)[c]**	366500 (297500-442000)	364500 (290500-479000)	0.785
**ESR (median (Q1-Q3), mm/hr)[c]**	63.00 (41.00-92.00)	72.00 (55.00-90.00)	0.304
**CRP (median (Q1-Q3), mg/L)[c]**	7.40 (3.49-15.13)	8.35 (3.79-13.19)	0.671
**GOT (median (Q1-Q3), IU/L)[c]**	34.00 (26.00-68.00)	37.00 (27.00-63.00)	0.671
**GPT (median (Q1-Q3), IU/L)[c]**	37.00 (18.00-101.00)	45.00 (20.00-112.00)	0.590
**CPK (median (Q1-Q3), IU/L)[c]**	41.00 (25.00-62.00)	47.00 (32.00-79.00)	0.152
**CPK/MB (median (Q1-Q3), ng/ml)[c]**	5.70 (3.60-7.30)	5.95 (3.85-7.70)	0.445
**Laboratory test (KD within 3-7 days after IVIG treatment)**			
**WBC (median (Q1-Q3), counts /μL)[c]**	11360.00 (8730.00-14500.00)	11260.00 (8065.00-13680.00)	0.445
**Hb (mean±SD, gm/dL)[b]**	11.00±1.28	10.96±1.64	0.872
**Hct (mean±SD, %)[b]**	32.20±3.57	32.64±4.84	0.540
**PLT (median (Q1-Q3), counts/μL)[c]**	554500.0 (462000.0-683000.0)	582000.0 (388000.0-702000.0)	0.981
**ESR (median (Q1-Q3), mm/hr)[c]**	77.00 (60.00-106.00)	87.00 (60.00-100.00)	0.527
**CRP (median (Q1-Q3), mg/L)[c]**	1.19 (0.53-4.33)	1.39 (0.78-5.68)	0.256
**GOT (median (Q1-Q3), IU/L)[c]**	42.50 (32.00-70.50)	40.00 (31.00-51.00)	0.470
**GPT (median (Q1-Q3), IU/L)[c]**	29.00 (21.00-68.00)	28.50 (22.00-65.00)	0.970
**CPK (median (Q1-Q3), IU/L)[c]**	34.50 (21.50-64.00)	40.00 (23.00-59.50)	0.626
**CPK/MB (median (Q1-Q3), ng/ml)[c]**	6.20 (3.90-9.40)	5.20 (2.20-6.00)	0.064

**Effective with 1st IVIG treatment**[a]			
**Yes (Number (%))**	176 (94.62%)	62 (81.58%)	***0.002***
**No (Number (%))**	10 (5.38%)	14 (18.42%)	
**Recurrence of KD**[a]			
**Yes (Number (%))**	2 (1.08%)	3 (3.95%)	0.148
**No (Number (%))**	184 (98.92%)	73 (96.05%)	

CAA, coronary artery aneurysm; IVIG, intravenous immunoglobulin; KD, kawasaki disease; WBC, white blood cells; Hb, hemoglobin; Hct, hematocrit; PLT, platelet; ESR, erythrocyte sedimentation rate; CRP: C-reactive protein; GOT: glutamic oxaloacetic transaminase ; GPT: glutamic-pyruvic transaminase; CPK, creatine phosphokinase; CPK/MB, creatine phosphokinase/myocardial band.

[a] Chi-square test.

[b] student-t test.

[c] Wilcoxon rank sum test.

Normal ranges for laboratory tests (WBC: 3.99-10.39 x 10^3/μL; Hb: 14.0-18.0 gm/dL for male; 12.0-16.0 gm/dL for female; Hct: 39-52 % for male; 35-48 % for female; PLT: 130-400 x 10^3/uL; ESR: 0-15 mm/hr for male; 0-20 mm/hr for female; CRP: less than 1.0 mg/L; GOT: 5-34 IU/L; GPT: 5-40 IU/L; CPK: 49-397 IU/L for male; 38-234 IU/L for female; CPK/MB: 0-3 ng/ml).

Bold, emphasizing statistical significance was considered as *p* value <0.05.

Table S2. Association results for the 203 SNPs that reached *p* < 0.005 in GWAS analysis of KD with CAA and without CAA

SNP	Chr.	Cytoband	Position[a]	Gene[b]	Gene Relationship	Genotype	*P*	OR
rs2411956	1	p36.32	4926409	MIR4417 / AJAP1	upstream / downstream	G/T	0.00146	2.719
rs11210499	1	p34.2	41624031	HIVEP3	intron	C/T	4.65E-04	0.3494

rs11210502	1	p34.2	41628040	HIVEP3	intron	A/C	7.32E-04	0.3658
rs2038977	1	p34.2	41628987	HIVEP3	intron	C/T	7.32E-04	0.3658
rs11807949	1	p34.2	41637205	HIVEP3	intron	C/T	1.77E-04	0.3157
rs4660316	1	p34.1	45807510	MAST2	intron	G/T	2.20E-04	3.119
rs3104449	1	p13.2	114822538	SIKE1 / SYCP1	upstream	C/G	1.89E-04	0.3148
rs12044828	1	p13.2	114981782	SYCP1	intron	C/T	2.25E-04	0.3279
rs11264793	1	q23.1	157677735	FCRL4 / FCRL3	upstream / downstream / UTR-3 / intron	A/T	3.21E-04	2.946
rs7549100	1	q23.1	157688051	FCRL3	intron	A/G	4.74E-04	2.826
rs10801121	1	q31.2	192509743	RGS1	upstream / downstream	A/G	3.80E-04	2.972
rs4658049	1	q31.2	192517982	RGS1 / RGS21	upstream / downstream / intron	A/T	5.48E-04	2.856
rs7537542	1	q32.1	199271032	LOC100131234 / NR5A2	upstream / intron	A/G	2.91E-04	0.3178
rs12095873	1	q32.1	202818914	LOC641515 / KDM5B-AS1	upstream / downstream	C/T	0.001924	2.649
rs1256623	1	q41	219087658	LOC643723	intron	C/G	0.001972	2.915
rs1146772	1	q41	219100066	LOC643723	intron	C/T	0.004042	2.733
rs6693436	1	q41	219112207	LOC643723	intron	C/T	0.001407	3.045
rs12119303	1	q41	219141207	LOC643723	intron	G/T	9.90E-04	3.193
rs12134260	1	q41	219141473	LOC643723	intron	C/T	0.001601	3.069
rs4846532	1	q41	219156014	LOC643723	intron	A/G	9.90E-04	3.193
rs6660443	1	q41	219159560	LOC643723	intron /// intron	C/T	0.002118	2.894
rs6541205	1	q41	219160887	LOC643723	intron	C/T	9.90E-04	3.193
rs10863436	1	q41	219194708	LYPLAL1	intron	A/G	0.00128	3.192
rs10746384	1	q41	219202204	LYPLAL1	intron	A/G	6.19E-04	3.373
rs10779342	1	q41	219206350	LYPLAL1	intron	A/T	0.001459	3.025
rs1568804	1	q41	219217862	RNU5F-1 / LYPLAL1	upstream / downstream	A/G	9.11E-04	3.216
rs6704354	1	q41	219220546	RNU5F-1 / LYPLAL1	upstream / downstream	C/T	9.11E-04	3.216
rs6670457	1	q41	219229084	RNU5F-1 / LYPLAL1	upstream / downstream	A/G	7.16E-04	3.335
rs11118243	1	q41	219233066	RNU5F-1 / LYPLAL1	upstream / downstream	C/T	7.16E-04	3.335
rs16848042	1	q42.13	227671682	ZNF678 / ZNF847P	downstream	C/G	4.20E-04	3.27
rs1923819	1	q42.13	227686612	ZNF678 / ZNF847P	downstream	C/T	6.45E-04	3.27
rs16848112	1	q42.13	227747048	SNAP47	intron	C/T	1.64E-05	4.075
rs6674275	1	q42.13	227748758	SNAP47	intron	C/T	1.64E-05	4.075
rs12064596	1	q42.13	227756257	SNAP47	intron	A/G	1.91E-05	4.033
rs12096217	1	q42.13	228346750	OBSCN	intron	A/G	0.001248	2.766

rs9701027	1	q42.13	229135163	RAB4A / RHOU	upstream / downstream	C/T	0.002016	2.505
rs9726077	1	q42.13	229136742	RAB4A / RHOU	upstream / downstream	A/G	0.002016	2.505
rs12072775	1	q42.13	229136805	RAB4A / RHOU	upstream / downstream	A/C	0.002016	2.505
rs12065106	1	q42.13	229138968	RAB4A / RHOU	upstream / downstream	G/T	0.002016	2.505
rs10916434	1	q42.13	229146263	RAB4A / RHOU	upstream /// downstream /// upstream /// downstream	C/T	0.002618	2.438
rs12064154	1	q42.13	229146328	RAB4A / RHOU	upstream / downstream	C/T	5.98E-04	2.76
rs10916435	1	q42.13	229150901	RAB4A / RHOU	upstream / downstream	C/G	8.54E-04	2.692
rs237784	1	q42.13	229335451	CCSAP	intron	A/G	6.21E-04	2.777
rs2345493	2	p24.2	18110934	KCNS3 / RDH14	downstream / intron	G/T	2.12E-05	4.971
rs2345496	2	p24.2	18135374	KCNS3 / RDH14	downstream / intron	A/G	1.60E-04	4.047
rs7573215	2	p21	45525000	SRBD1	intron	C/T	4.37E-04	2.9
rs17033801	2	p21	45531887	SRBD1	intron	A/C	4.87E-04	2.866
rs3755082	2	p21	45574259	SRBD1	intron	C/T	5.95E-04	2.825
rs10202102	2	p13.3	68784021	ARHGAP25	intron	C/T	3.65E-04	3.656
rs17022036	2	q11.2	98880464	C2orf55	intron	G/T	7.82E-04	2.701
rs10174913	2	q14.1	116017590	DDX18 / DPP10	upstream / downstream	A/C	5.01E-04	3.945
rs10196916	2	q14.1	116042817	DDX18 / DPP10	upstream / downstream	C/G	5.83E-04	3.881
rs16831039	2	q33.1	199144083	PLCL1 / SATB2	downstream	C/T	8.51E-05	3.31
rs1376584	2	q33.1	199144170	PLCL1 / SATB2	downstream	C/T	8.51E-05	3.31
rs1901321	2	q33.1	199148856	PLCL1 / SATB2	downstream	A/G	5.42E-05	3.472
rs1451473	2	q33.1	199150370	PLCL1 / SATB2	downstream / upstream	G/T	1.17E-04	3.23
rs921465	2	q33.1	199150704	PLCL1 / SATB2	downstream	A/C	7.39E-05	3.391
rs1868913	2	q33.1	199162581	PLCL1 / SATB2	downstream	A/T	8.51E-05	3.31
rs1901323	2	q33.1	199162766	PLCL1 / SATB2	downstream / upstream	A/T	8.51E-05	3.31
rs10931853	2	q33.1	199163235	PLCL1 / SATB2	downstream // upstream	C/T	8.51E-05	3.31
rs6730991	2	q33.1	199165025	PLCL1 / SATB2	downstream / upstream	A/G	8.51E-05	3.31
rs16831114	2	q33.1	199166663	PLCL1 / SATB2	downstream /	C/T	9.34E-05	3.288

					upstream			
rs1584661	2	q33.1	199170501	PLCL1 / SATB2	downstream / upstream	C/T	8.51E-05	3.31
rs6742079	2	q33.1	199173285	PLCL1 / SATB	downstream / upstream	A/G	8.51E-05	3.31
rs6717968	2	q33.1	199193218	PLCL1 / SATB2	downstream	C/T	8.51E-05	3.31
rs10804088	2	q33.1	199203328	PLCL1 / SATB2	downstream	A/G	8.51E-05	3.31
rs1376591	2	q33.1	199203436	PLCL1 / SATB2	downstream	C/T	8.51E-05	3.31
rs4338918	2	q33.1	199208128	PLCL1 / SATB2	downstream	A/T	8.51E-05	3.31
rs12616812	2	q33.1	199208466	PLCL1 / SATB2	downstream	C/T	1.24E-04	3.229
rs11885518	2	q36.3	229336180	PID1 / DNER	upstream / downstream	C/G	8.54E-04	3.748
rs11695484	2	q37.1	233745802	UGT1A8 / UGT1A10 / UGT1A9 / UGT1A7 / UGT1A6 / UGT1A5 / UGT1A4 / UGT1A3/ UGT1A7	intron	A/G	4.90E-04	0.2109
rs887829	2	q37.1	233759923	UGT1A8 / UGT1A10 / UGT1A9 / UGT1A7 / UGT1A6 / UGT1A5 / UGT1A4 / UGT1A3/ UGT1A7	intron	A/G	4.35E-04	0.208
rs6742078	2	q37.1	233763992	UGT1A8 / UGT1A10 / UGT1A9 / UGT1A7 / UGT1A6 / UGT1A5 / UGT1A4 / UGT1A3/ UGT1A7	intron	G/T	4.90E-04	0.2109
rs6796318	3	p22.3	32260293	CMTM8	intron	A/G	7.32E-04	3.415
rs4132830	3	p22.3	32273890	CMTM8	intron	C/T	0.002041	3.058
rs7633253	3	p12.3	78610399	ROBO1	intron	C/T	0.002973	0.3984
rs3821595	3	p12.3	78619433	ROBO1	intron	A/T	0.001671	0.3807
rs6807510	3	p12.3	78630434	ROBO1	intron	C/T	0.001684	0.3808
rs13092536	3	q11.2	96076517	EPHA6 / LOC255025 / LOC100287639	upstream / downstream	A/G	8.13E-04	0.2873
rs4416398	3	q11.2	96092537	EPHA6 / LOC255025 / LOC100287639	upstream / downstream	C/G	5.67E-04	0.2633
rs13085875	3	q11.2	96106634	EPHA6 / LOC255025 / LOC100287639	upstream / downstream	A/G	7.25E-04	0.2837
rs9838562	3	q13.13	111302133	CD96 / PVRL3	upstream / downstream / intron	A/C	8.58E-04	0.3696
rs6793657	3	q21.3	127185449	PLXNA1 / TPRA1 / C3orf56	downstream / upstream	C/T	3.73E-04	0.2914
rs13072025	3	q21.3	127199552	PLXNA1 / TPRA1 / C3orf56	downstream	A/T	7.91E-04	0.3694
rs11923216	3	q21.3	127205138	PLXNA1 / TPRA1	downstream	C/T	7.91E-04	0.3694
rs10015056	4	p15.1	35182075	PCDH7 / ARAP2	downstream / upstream	A/G	0.001788	0.3826
rs2411265	4	q12	52777311	ERVMER34-1 / LOC152578	upstream	A/G	0.00185	2.517
rs17613967	4	q12	52779752	ERVMER34-1 / LOC152578	upstream	G/T	0.001459	2.571

rs10028567	4	q12	52791409	LOC152578	intron	C/T	0.001459	2.571
rs13122232	4	q12	53069099	SCFD2	intron	A/G	3.03E-04	0.3172
rs4327488	4	q24	101738055	BANK1 / FLJ20021	upstream / downstream / intron	C/T	4.08E-04	2.961
rs1499331	4	q28.3	132357757	PCDH10 / C4orf33	upstream / downstream	A/G	0.002605	2.891
rs4862161	4	q35.1	183349528	CLDN24 / CDKN2AIP	upstream / downstream	C/T	4.35E-04	0.2932
rs17608672	5	p15.33	2645450	IRX4 / IRX2	upstream / downstream	C/T	0.001117	3.916
rs9313144	5	p15.32	6132052	KIAA0947 / FLJ33360	downstream / upstream	C/T	4.19E-05	0.2777
rs2966959	5	p14.3	19370608	LOC401177 / CDH18	upstream / downstream	A/G	4.29E-05	0.2865
rs10519436	5	q23.1	115987791	AQPEP	intron	C/T	5.39E-04	0.344
rs718734	5	q23.1	120074232	PRR16 / FAM170A	upstream / downstream	A/T	0.001536	0.3928
rs12514641	5	q23.1	120075328	PRR16 / FAM170A	upstream / downstream	C/T	9.74E-04	0.3764
rs10045387	5	q23.1	120076291	PRR16 / FAM170A	upstream / downstream / UTR-3 / intron	A/G	6.86E-04	0.3629
rs10066059	5	q23.1	120095375	PRR16 / FAM170A	upstream / downstream	C/T	0.001711	0.393
rs10042286	5	q23.2	126097099	ZNF608 / GRAMD3	upstream / intron	A/G	0.001124	3.076
rs356486	5	q31.2	139705517	PSD2 / CXXC5	upstream / downstream / intron	C/G	0.002847	2.624
rs29808	5	q35.1	169576803	SLIT3 / CCDC99	upstream / downstream	A/G	0.001239	0.3831
rs29807	5	q35.1	169577544	SLIT3 / CCDC99	upstream / downstream	A/G	0.001239	0.3831
rs17668965	5	q35.1	169590225	CCDC99	intron	A/G	2.09E-04	3.032
rs2317217	6	p25.3	796483	EXOC2 / LOC285768	upstream / downstream	A/C	2.05E-04	0.3254
rs7757332	6	p25.3	800495	EXOC2 / LOC285768	upstream / downstream	C/T	0.001312	0.3595
rs9328211	6	p25.2	3989419	PRPF4B / FAM50B	upstream / downstream	A/G	2.67E-04	0.321
rs688176	6	p25.2	4067874	PRPF4B / FAM217A	downstream / intron	C/T	1.51E-04	0.3233
rs593291	6	p25.2	4068481	FAM217A	UTR-3 / intron / exon	A/C	1.56E-04	0.3187
rs595413	6	p25.2	4068931	FAM217A	missense / intron //	C/T	1.32E-04	0.3189

					cds / exon			
rs2783063	6	p25.2	4080643	C6orf201 / FAM217A	intron	G/T	5.27E-04	0.3568
rs11755877	6	p25.2	4081942	C6orf201 / FAM217A	intron	C/G	3.50E-04	0.347
rs101418	6	p25.2	4083037	C6orf201 / FAM217A	intron	C/T	1.38E-04	0.3213
rs662834	6	p25.2	4083154	C6orf201 / FAM217A	intron	C/G	3.50E-04	0.347
rs634114	6	p25.2	4086685	C6orf201 / FAM217A	intron	C/G	1.38E-04	0.3213
rs707991	6	p25.2	4097677	C6orf201	intron	A/G	1.04E-04	0.3145
rs3132551	6	p21.33	31193861	PSORS1C1 / CDSN	intron / exon	C/T	6.50E-04	2.85
rs6920036	6	q12	65106985	EYS	intron	A/G	0.001262	3.16
rs6917799	6	q12	65109261	EYS	intron	G/T	0.001262	3.16
rs9294634	6	q12	65116653	EYS	intron	G/T	0.001262	3.16
rs9453188	6	q12	65117743	EYS	intron	G/T	0.001262	3.16
rs16896290	6	q12	65118594	EYS	intron	G/T	8.21E-04	3.272
rs11962884	6	q12	65150645	EYS	intron	A/G	7.87E-04	3.139
rs16896391	6	q12	65196104	EYS	intron	C/T	0.001035	3.065
rs16896392	6	q12	65196285	EYS	intron	C/T	8.71E-04	3.104
rs841531	6	q12	65196436	EYS	intron	C/T	6.85E-04	3.196
rs10485313	6	q12	65210760	EYS	intron	A/G	6.03E-04	3.241
rs539248	6	q12	65218659	EYS	intron	A/T	6.57E-04	3.291
rs4142063	6	q12	65259036	EYS	intron	G/T	2.30E-04	3.609
rs6915695	6	q12	65269380	EYS	intron	A/T	2.30E-04	3.609
rs4991400	6	q13	73210711	KCNQ5 / KHDC1L	downstream / intron	A/T	3.83E-04	0.3496
rs6453655	6	q13	73210829	KCNQ5 / KHDC1L	downstream / intron	A/T	3.83E-04	0.3496
rs2107806	7	p21.3	10246934	PER4 / NDUFA4	downstream / upstream	C/G	0.001702	0.3966
rs3960052	7	p21.3	10248605	PER4 / NDUFA4	downstream / upstream	A/G	5.99E-04	0.3517
rs10265578	7	p21.3	10254293	PER4 / NDUFA4	downstream / upstream	C/T	7.87E-04	0.3682
rs706080	7	p21.1	16808153	AGR2 / AGR3	upstream / downstream / intron	A/G	0.002296	0.4099
rs2008296	7	p15.1	28105944	JAZF1	intron	C/T	0.001663	0.3614
rs6977955	7	p15.1	28117267	JAZF1	intron	C/T	0.001309	0.3535
rs10951192	7	p15.1	28121413	JAZF1	intron	C/G	0.001309	0.3535
rs11495981	7	p15.1	28137681	JAZF1	intron	C/T	0.001195	0.3493
rs864745	7	p15.1	28140936	JAZF1	intron	A/G	5.86E-04	0.287
rs41420446	7	p15.1	28177760	JAZF1	intron	A/G	4.70E-04	0.2814
rs1868651	7	p14.1	37601817	ELMO1 / GPR141	upstream	G/T	5.35E-04	0.3538
rs7795852	7	q34	140300704	LOC100134229 / SLC37A3	downstream / intron	A/G	8.94E-05	3.768
rs7341537	7	q35	146883620	CNTNAP2	intron	A/C	0.001902	0.3765
rs882794	7	q36.1	149445637	ZNF777	intron	A/G	8.83E-04	0.368
rs6993670	8	p22	18871430	PSD3	intron	A/G	1.88E-05	4.184

rs13265893	8	q11.23	54252174	SOX17 / MRPL15 / RNU105C	upstream / downstream	C/T	8.70E-04	2.688
rs10781028	9	q21.13	71554026	TRPM3 / TMEM2	upstream / downstream	C/T	3.78E-04	3.05
rs10119687	9	q22.33	97710546	XPA / FOXE1	upstream / downstream	A/G	8.14E-05	3.555
rs1016428	9	q31.1	101639252	GRIN3A	intron	C/T	0.001392	0.3705
rs7849782	9	q31.1	101664981	GRIN3A	intron	C/G	7.54E-06	0.2482
rs10906943	10	q11.22	46301763	ANXA8L2 / FAM35B2	upstream / downstream / intron	A/G	0.002605	0.4023
rs10740217	10	q21.3	66025418	CTNNA3	intron	A/T	1.51E-04	3.132
rs912745	10	q26.11	117738648	EMX2 / RAB11FIP2	downstream	C/T	2.43E-04	4.945
rs11016509	10	q26.3	128837321	MKI67 / MGMT	upstream / downstream	C/T	0.002837	2.789
rs217756	11	p15.1	16784612	C11orf58 / PLEKHA7	downstream / intron	G/T	9.84E-05	4.557
rs7126354	11	q12.2	60463592	MS4A1	intron	C/T	0.001369	2.904
rs2450908	11	q13.2	68254537	SUV420H1 / C11orf24	upstream / downstream	C/T	2.70E-04	0.3323
rs538079	11	q23.3	117471504	DSCAML1	intron	A/G	7.79E-04	0.3579
rs520289	11	q23.3	117479421	DSCAML1	intron	A/G	5.12E-04	0.345
rs11827507	11	q25	132164575	NTM	intron	A/G	0.002371	2.545
rs3962605	12	p13.1	13125501	GSG1 / EMP1 / KIAA1467	upstream / intron	C/T	3.68E-04	3.74
rs926150	12	p11.22	28087892	PTHLH / CCDC91	upstream	C/G	7.79E-04	0.3701
rs1073083	12	q23.2	101701161	CHPT1	intron	A/T	0.001578	0.373
rs470393	12	q24.33	128972744	GLT1D1	intron	C/T	4.00E-04	3.818
rs2324129	13	q13.3	39107894	LHFP / NHLRC3	downstream	A/G	0.002132	2.79
rs12590437	14	q21.1	37977886	FOXA1 / SSTR1 / LOC100652860	upstream / intron	A/T	9.31E-06	4.009
rs1959674	14	q21.1	37999337	FOXA1 / SSTR1 / LOC100652860	upstream / intron	A/G	0.00425	2.864
rs41356944	14	q21.1	37999387	FOXA1 / SSTR1 / LOC100652860	upstream / intron	C/T	0.004738	2.818
rs17127854	14	q22.2	54737938	SAMD4A	intron	C/G	1.97E-04	3.066
rs10144855	14	q22.2	54745523	SAMD4A	intron	C/G	1.34E-04	3.159
rs17774131	16	q22.1	69469883	CYB5B / MIR1538 / NFAT5	downstream / upstream	C/G	0.001114	2.674
rs3826139	16	q23.3	84081673	MBTPS1	intron	C/T	0.001896	0.3995
rs7200337	16	q23.3	84086209	MBTPS1	intron	C/G	0.001689	0.3959
rs12598083	16	q24.1	86397391	LOC732275 / FOXF1-AS1	upstream / downstream	C/G	2.90E-05	3.615
rs11077622	17	q24.3	72676523	SLC39A11	intron	A/G	3.61E-04	0.2998
rs11077623	17	q24.3	72676560	SLC39A11	intron	C/G	3.61E-04	0.2998
rs9900792	17	q25.3	78411544	PGS1	intron	C/T	0.001913	2.568
rs11082212	18	q12.3	40803209	LOC647946 / KC6	upstream / downstream	A/G	6.96E-05	4.123

rs1431301	18	q12.3	40818374	LOC647946 / KC6	upstream / downstream	A/G	6.96E-05	4.123
rs2313647	18	q12.3	40819127	LOC647946 / KC6	upstream / downstream	C/T	6.96E-05	4.123
rs17756653	18	q12.3	40820189	LOC647946 / KC6	upstream / downstream	C/T	6.96E-05	4.123
rs323585	18	q12.3	40865874	LOC647946 / KC6	upstream / downstream	A/G	1.08E-04	3.906
rs2567173	18	q12.3	40867137	LOC647946 / KC6	upstream / downstream	C/T	0.001926	3.117
rs8087876	18	q12.3	44435695	SYT4 / SETBP1	upstream / downstream	G/T	0.002413	0.4112
rs9961416	18	q12.3	44457227	SYT4 /// SETBP1	upstream / downstream	A/G	0.002644	0.4125
rs9951264	18	q12.3	44457712	SYT4 / SETBP1	upstream / downstream	G/T	0.00149	0.3937
rs3829634	18	q21.33	62043093	RNF152 / PIGN	upstream / downstream	C/T	9.56E-04	2.775
rs9965630	18	q21.33	62065040	PIGN	intron	A/G	7.53E-04	2.833
rs1030583	18	q21.33	62068449	PIGN	intron	C/G	1.88E-04	3.526
rs10503065	18	q21.33	62124286	PIGN	intron	A/G	7.77E-04	3.792
rs11659253	18	q23	77895725	SALL3 / GALR1	upstream / downstream	C/T	2.52E-04	3.343
rs6140791	20	p12.3	8906575	PLCB4 / PLCB1	upstream / downstream / intron	C/G	4.08E-04	2.861
rs6086686	20	p12.3	8909249	PLCB4 / PLCB1	upstream / downstream / intron	A/G	7.39E-04	2.712
rs2179798	20	p12.3	8910698	PLCB4 / PLCB1	upstream / downstream / intron	A/G	0.001633	2.528
rs962348	20	p12.3	8910988	PLCB4 / PLCB1	upstream / downstream / intron	C/G	0.001328	2.575
rs6140795	20	p12.3	8916834	PLCB4 / PLCB1	upstream / downstream / intron	C/T	9.06E-04	2.664
rs4299396	20	p12.3	8934667	PLCB4 / PLCB1	upstream / downstream / intron	A/T	2.12E-04	3.057
rs16995415	20	p12.3	8935154	PLCB4 / PLCB1	upstream / downstream / intron	A/G	2.72E-04	2.962
rs16986312	20	q12	41940309	CHD6 / PTPRT	upstream / downstream	A/G	0.002701	0.3187

Association results are ordered by the chromosome, cytoband, and

position.

[a]Chromosome positions are based on NCBI GRCh38 version.

[b]Defined as the gene containing the SNP or the closest genes (within 100 kb up- and downstream) to the SNP.

SNP, single nucleotide polymorphism; CAA, KD patients with coronary artery aneurysm; OR, odds ratio.

Table S3. Top networks associated with CAA in KD

ID	Top Diseases and Functions	Score	Focus Molecules	Contributing genes
**1**	Embryonic Development, Nervous System Development and Function, Organ Development, Lipid Metabolism	40	19	*Calmodulin,caspase,Cg,CMTM8,EMP1,EPHA6,ERK,ERK1/2,EXOC2,FSH,Gpcr,GTPase,Histone h3,HIVEP3,Insulin,KCNQ5,KHDC1L,MAST2,MGMT,MS4A1,NFkB (complex),NR5A2,PID1,Pkc(s),PLC,PLCB1,PLCB4,PLCL1,PSD3,RAB4A,ROBO1,SLIT3,SOX17,TRPM3,Vegf*
**2**	Lipid Metabolism, Small Molecule Biochemistry, Vitamin and Mineral Metabolism	28	16	*CDH18,CDKN2AIP,CEPT1,CHD6,CNTNAP2,CYB5B,DHRS3,ELMO1,ELMO2,ELMO3,FARP2,KIAA1467,KNTC1,NBAS,NT5C1B,PIGG,PIGN,PIGO,PLEKHA7,PLEKHG6,PVRL3,RAD21L1,RDH10,RDH11,RDH12,RDH14,SCFD2,SDR16C5,SIKE1,SPDL1,SRBD1,SYCP1,TSEN54,UBC,ZWILCH*
**3**	Cell Morphology, Cellular Assembly and	26	14	*ARHGAP25,ARHGDIB,CCDC91,CCSAP,CDSN,CEPT1,CHPT1,CPSF6,DRD4,GGA1,GGA2,JAZF1,KDM5B-AS1,KLHL12,KLK5,KLK7,LYPLAL1,NDC80,NHLRC3,PDLIM1,PEF1,PGS1,POF1B,RAG2,SNAP47,TAOK2,TESC,TPRA1,UBC,UBXN1,UBXN7,VTA1,ZNF608,ZNF678,ZNF777*

**4**	Organization, Dermatological Diseases and Conditions Cellular Movement, Reproductive System Development and Function, Cell Morphology	24	12	*AGR3,APP,C11orf24,C6orf201,CCIN,DNAJC4,DNAJC12,DSCAML1,FAM127A,FAM127B,FBXL13,GLT1D1,GSG1,Hsp84-2,HSP90AA1,KCNS3,KLHL32,MRPL15,NEK11,NPHP4,NTM,OBSCN,PRR16,RGS1,SETBP1,SPEM1,SPZ1,SYT4,TAF7L,TBX22,THAP4,TPM1,TSKS,TSSK4,UBQLN1*

Table S4. Genetic variants in PLCB4 and PLCB1 associated with phenotype traits reported in PheGen website.

#	Trait	SNP (rs number)	Context	Gene	Gene ID	Gene 2	Gene ID 2	Chromosome	Location	P-Value	Source	PubMed
1	Leukocyte Count	2072910	intron	PLCB4	5332	PLCB4	5332	20	9365303	3.00E-10	NHGRI	20172861
2	Psychomotor Performance	6056209	intron	PLCB1	23236	PLCB1	23236	20	8836771	2.00E-06	NHGRI	19734545
3	Body Weight	10485729	intron	PLCB4	5332	PLCB4	5332	20	9165158	2.19E-06	dbGaP	17903300
4	Body Weight	10485729	intron	PLCB4	5332	PLCB4	5332	20	9165158	2.19E-06	dbGaP	17903300
5	Body Weight Changes	1014827	intron	PLCB1	23236	PLCB1	23236	20	8751162	2.90E-06	dbGaP	17903300
6	Body Weight Changes	1014827	intron	PLCB1	23236	PLCB1	23236	20	8751162	2.90E-06	dbGaP	17903300
7	Apolipoproteins B	6133598	intron	PLCB1	23236	PLCB1	23236	20	8649730	6.43E-06	dbGaP	17903299
8	Apolipoproteins B	6133598	intron	PLCB1	23236	PLCB1	23236	20	8649730	6.43E-06	dbGaP	17903299
9	Body Weight	10485729	intron	PLCB4	5332	PLCB4	5332	20	9165158	8.39E-06	dbGaP	17903300
10	Body Weight	10485729	intron	PLCB4	5332	PLCB4	5332	20	9165158	8.39E-06	dbGaP	17903300
11	Platelet Count	2179321	intron	PLCB4	5332	PLCB4	5332	20	9371825	1.42E-05	dbGaP	0
12	Platelet Count	2179321	intron	PLCB4	5332	PLCB4	5332	20	9371825	1.42E-05	dbGaP	0
13	Body Mass Index	10485729	intron	PLCB4	5332	PLCB4	5332	20	9165158	2.23E-05	dbGaP	17903300
14	Body Mass Index	10485729	intron	PLCB4	5332	PLCB4	5332	20	9165158	2.23E-05	dbGaP	17903300
15	Platelet Count	725941	intron	PLCB4	5332	PLCB4	5332	20	9349033	2.35E-05	dbGaP	0
16	Platelet Count	725941	intron	PLCB4	5332	PLCB4	5332	20	9349033	2.35E-05	dbGaP	0
17	Body Weight	10485729	intron	PLCB4	5332	PLCB4	5332	20	9165158	2.61E-05	dbGaP	17903300
18	Body Weight	10485729	intron	PLCB4	5332	PLCB4	5332	20	9165158	2.61E-05	dbGaP	17903300
19	Body Weight	10485729	intron	PLCB4	5332	PLCB4	5332	20	9165158	2.69E-05	dbGaP	17903300
20	Body Weight	10485729	intron	PLCB4	5332	PLCB4	5332	20	9165158	2.69E-05	dbGaP	17903300
21	Body Mass Index	10485729	intron	PLCB4	5332	PLCB4	5332	20	9165158	2.75E-05	dbGaP	17903300
22	Body Mass Index	10485729	intron	PLCB4	5332	PLCB4	5332	20	9165158	2.75E-05	dbGaP	17903300
23	Body Mass Index	10485729	intron	PLCB4	5332	PLCB4	5332	20	9165158	3.53E-05	dbGaP	17903300
24	Body Mass Index	10485729	intron	PLCB4	5332	PLCB4	5332	20	9165158	3.53E-05	dbGaP	17903300
25	Body Mass Index	10485729	intron	PLCB4	5332	PLCB4	5332	20	9165158	5.77E-05	dbGaP	17903300

26	Body Mass Index	10485729	intron	PLCB4	5332	PLCB4	5332	20	9165158	5.77E-05	dbGaP	17903300
27	Body Mass Index	10485729	intron	PLCB4	5332	PLCB4	5332	20	9165158	6.00E-05	dbGaP	17903300
28	Body Mass Index	10485729	intron	PLCB4	5332	PLCB4	5332	20	9165158	6.00E-05	dbGaP	17903300
29	Cholesterol, HDL	6056625	intron	PLCB4	5332	PLCB4	5332	20	9420614	6.10E-05	dbGaP	17903299
30	Cholesterol, HDL	6056625	intron	PLCB4	5332	PLCB4	5332	20	9420614	6.10E-05	dbGaP	17903299
31	Cholesterol, HDL	7265537	intron	PLCB4	5332	PLCB4	5332	20	9368554	6.80E-05	dbGaP	0
32	Cholesterol, HDL	7265537	intron	PLCB4	5332	PLCB4	5332	20	9368554	6.80E-05	dbGaP	0
33	Tunica Media	8115510	intron	PLCB4	5332	PLCB4	5332	20	9313941	6.86E-05	dbGaP	0
34	Tunica Media	8115510	intron	PLCB4	5332	PLCB4	5332	20	9313941	6.86E-05	dbGaP	0
35	Body Weight	10485729	intron	PLCB4	5332	PLCB4	5332	20	9165158	6.95E-05	dbGaP	17903300
36	Body Weight	10485729	intron	PLCB4	5332	PLCB4	5332	20	9165158	6.95E-05	dbGaP	17903300
37	Tunica Media	7268671	intron	PLCB4	5332	PLCB4	5332	20	9322989	7.78E-05	dbGaP	0
38	Tunica Media	7268671	intron	PLCB4	5332	PLCB4	5332	20	9322989	7.78E-05	dbGaP	0
39	Body Weight	10485729	intron	PLCB4	5332	PLCB4	5332	20	9165158	9.50E-05	dbGaP	17903300
40	Body Weight	10485729	intron	PLCB4	5332	PLCB4	5332	20	9165158	9.50E-05	dbGaP	17903300
41	Parkinson Disease	6039424	intron	PLCB4	5332	PLCB4	5332	20	9225164	9.55E-05	dbGaP	16252231
42	Stroke	2808806	intergenic	RNU105B	26767	PLCB4	5332	20	8942998	0.0005656	dbGaP	0
43	Stroke	10485724	intron	PLCB1	23236	PLCB1	23236	20	8456324	0.0009041	dbGaP	0

PheGen website: http://www.ncbi.nlm.nih.gov/gap/phegeni

Figure S1

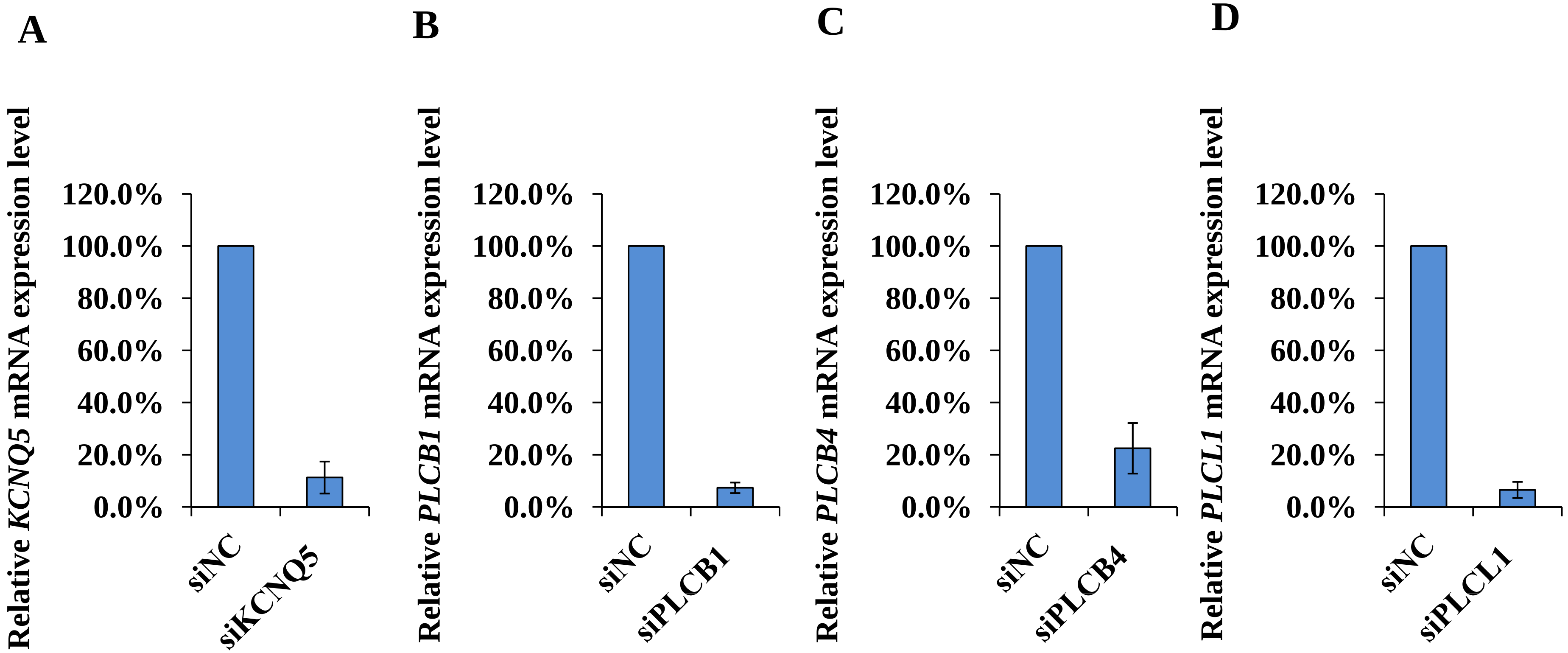

Figure S1. RT-qPCR analysis for *KCNQ5*, *PLCB1*, *PLCB4* and *PLCL1* genes by siRNAs down-regulation technigue. HUVEC cells were transfected with siRNAs or siNC for 24 h at 37°C followed by 100 μg/mL LPS for another 24 h. A, *KCNQ5* mRNA expression. B, *PLCB1* mRNA expression. C, *PLCB4* mRNA expression. D, *PLCL1* mRNA expression. Values are normalized to those of siNC-transfected cells. Data represent mean ± SD for three independent experiments.

Figure S2

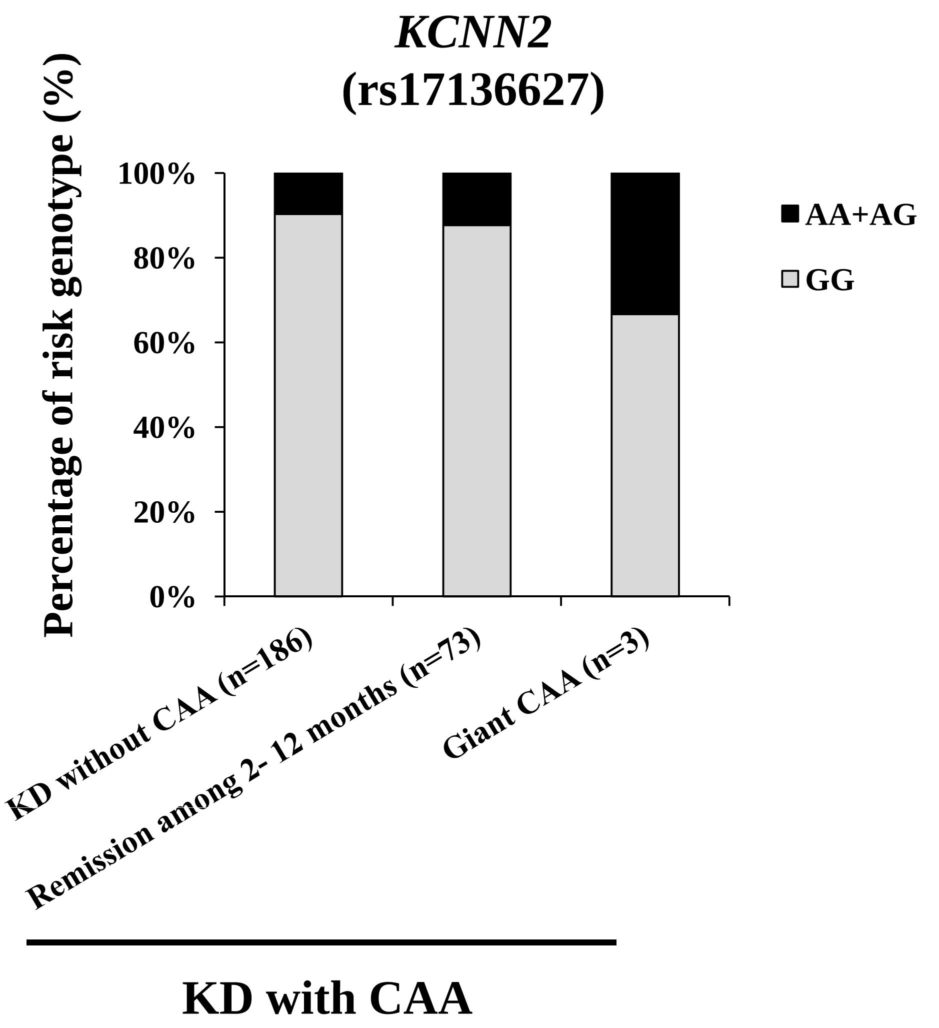

Figure S2. The distribution of the risk genotype frequency of rs17136627 in the *KCNN2* gene according to the CAA severity. CAA was identified when either the right or the left coronary artery showed an increase in the dilated diameter by > 3 mm in children below 5 years of age or by > 4 mm in older children. CAA severity grade: KD without CAA indicates patients with no CAA complications; KD with CAA (remission between 2-12 months) indicates patients with CAA, but who showed remission between 2-12 months after KD illness; KD with CAA (giant CAA) indicates patients with giant CAA(≥ 8mm) or severe stenosis or occlusion. The genotypes of rs17136627 were shown by the CAA severity.

Figure S3

A

rs6857404

Study name	Odds ratio	Lower limit	Upper limit	Z-Value	p-Value
Kim JJ et al., 2013	8.780	2.863	26.927	3.800	0.000
Our study in Taiwan	1.571	0.693	3.561	1.082	0.279
	3.553	0.659	19.141	1.475	0.140

Statistics for each study

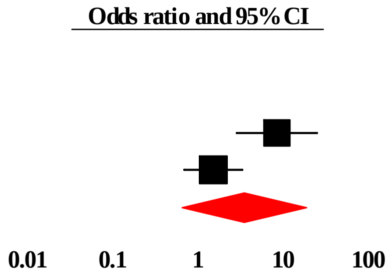

B

rs17136627

Study name	Odds ratio	Lower limit	Upper limit	Z-Value	p-Value
Kim JJ et al., 2013	12.600	4.127	38.470	4.449	0.000
Our study in Taiwan	1.377	0.568	3.339	0.708	0.479
	4.054	0.463	35.463	1.265	0.206

Statistics for each study

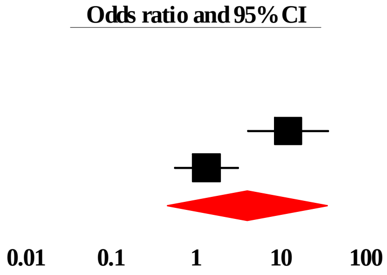

C

rs189517

Study name	Odds ratio	Lower limit	Upper limit	Z-Value	p-Value
Kim JJ et al., 2013	8.850	2.885	27.151	3.812	0.000
Our study in Taiwan	0.517	0.192	1.392	-1.305	0.192
	2.112	0.131	34.154	0.527	0.598

Statistics for each study

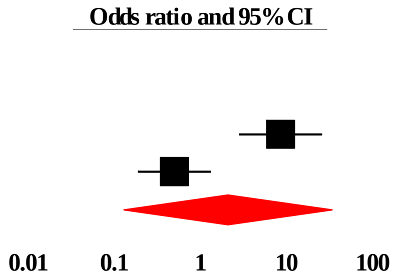

D

rs13248977

Study name	Odds ratio	Lower limit	Upper limit	Z-Value	p-Value
Kim JJ et al., 2013	6.420	2.604	15.826	4.039	0.000
Our study in Taiwan	0.870	0.340	2.226	-0.291	0.771
	2.374	0.335	16.830	0.865	0.387

Statistics for each study

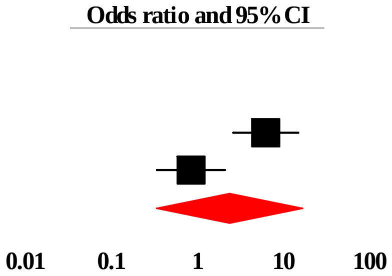

Figure S3. Meta-analysis of the association of the previously reported CAA-associated loci, the rs6857404, rs17136627, rs189517, and rs13248977 with KD in a case-control design study including a Korean ancestry and a Taiwanese ancestry. A, Forest plot showing-the SNP-rs6857404 for odds ratio and its 95% CI plotted with a box and a horizontal line. Random-effects pooled odds ratio=3.553, 95% CI=0.659-19.141, *p*=0.140. B, Forest plot showing-the SNP-rs17136627 for odds ratio and its 95% CI plotted with a box and a horizontal line. Random-effects pooled odds ratio=4.054, 95% CI=0.463-35.463, *p*=0.206. C, Forest plot showing-the SNP- rs189517 for odds ratio and its 95% CI plotted with a box and a horizontal line. Random-effects pooled odds ratio=2.112, 95% CI=0.131-34.154, *p*=0.598. D, Forest plot showing-the SNP-rs13248977 for odds ratio and its 95% CI plotted with a box and a horizontal line. Random-effects pooled odds ratio=2.374, 95% CI=0.335-16.830, *p*=0.387.

Figure S4

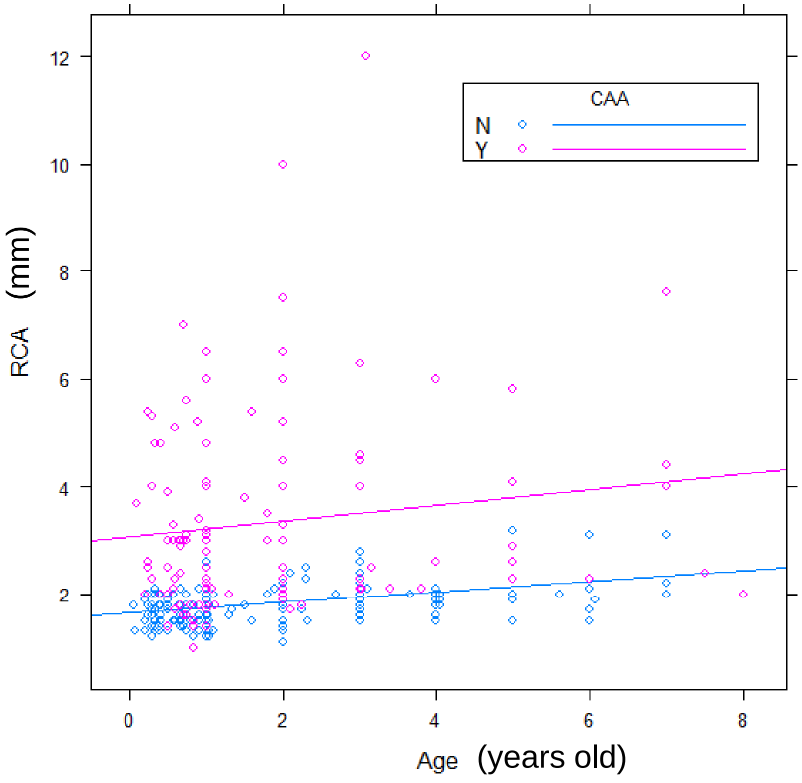

Figure S4. Right coronary artery (RCA) diameter verus age. KD with CAA group were with red line. KD without CAA group were with blue line.

Figure S5

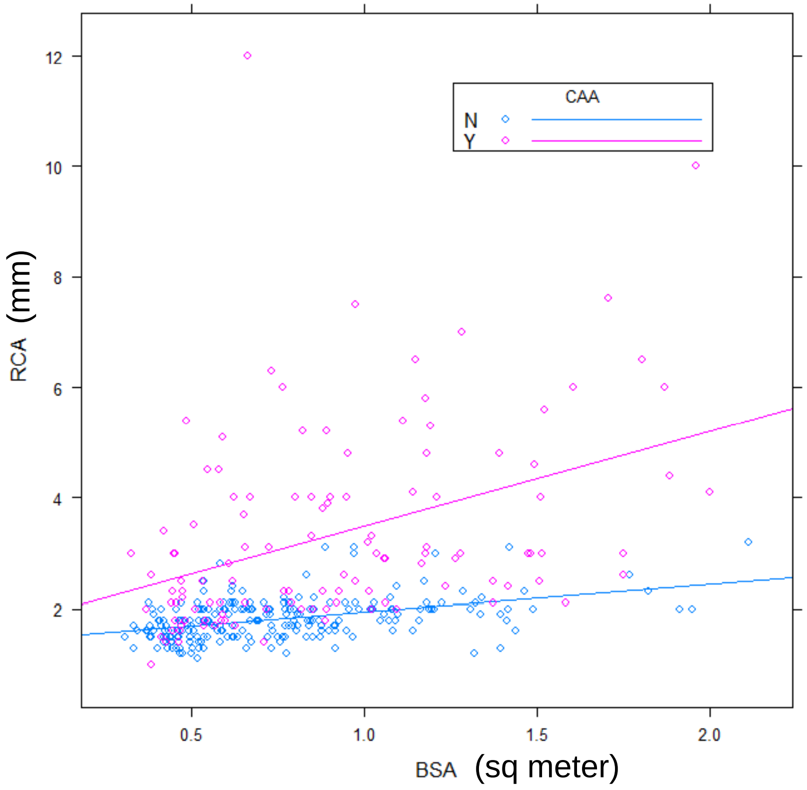

Figure S5. Right coronary artery (RCA) diameter verus body surface area. KD with CAA group were with red line. KD without CAA group were with blue line.

Figure S6

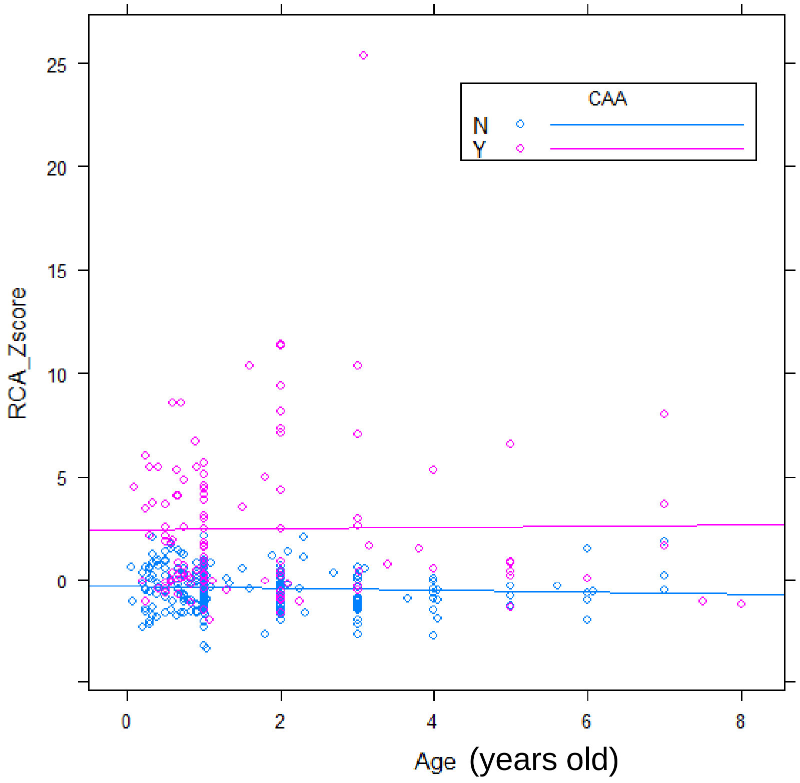

Figure S6. Right coronary artery (RCA) Z-score verus age. KD with CAA group were with red line. KD without CAA group were with blue line.

Figure S7

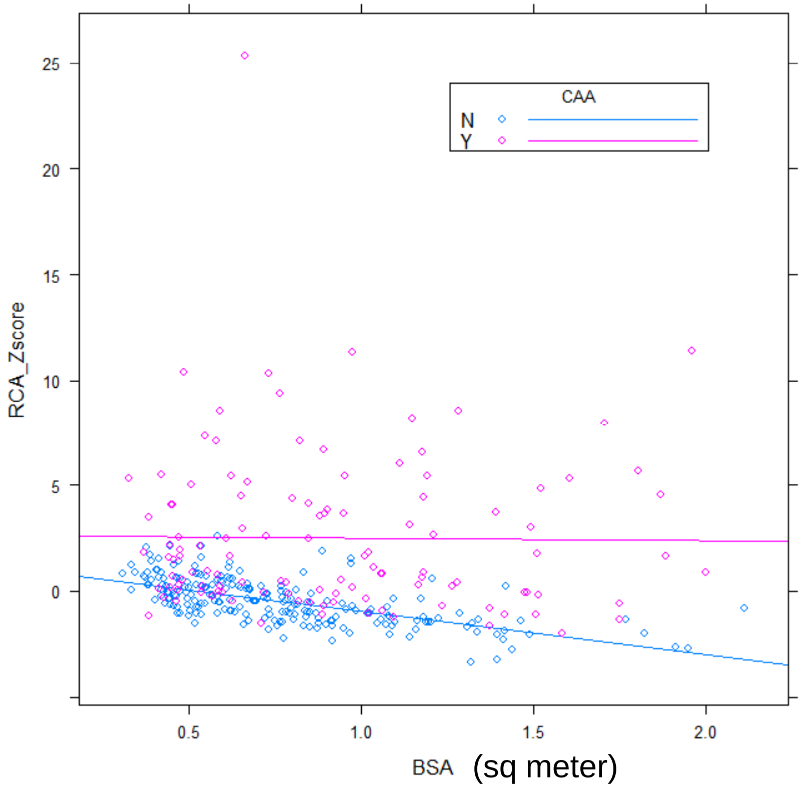

Figure S7. Right coronary artery (RCA) Z-score verus body surface area. KD with CAA group were with red line. KD without CAA group were with blue line.

Figure S8

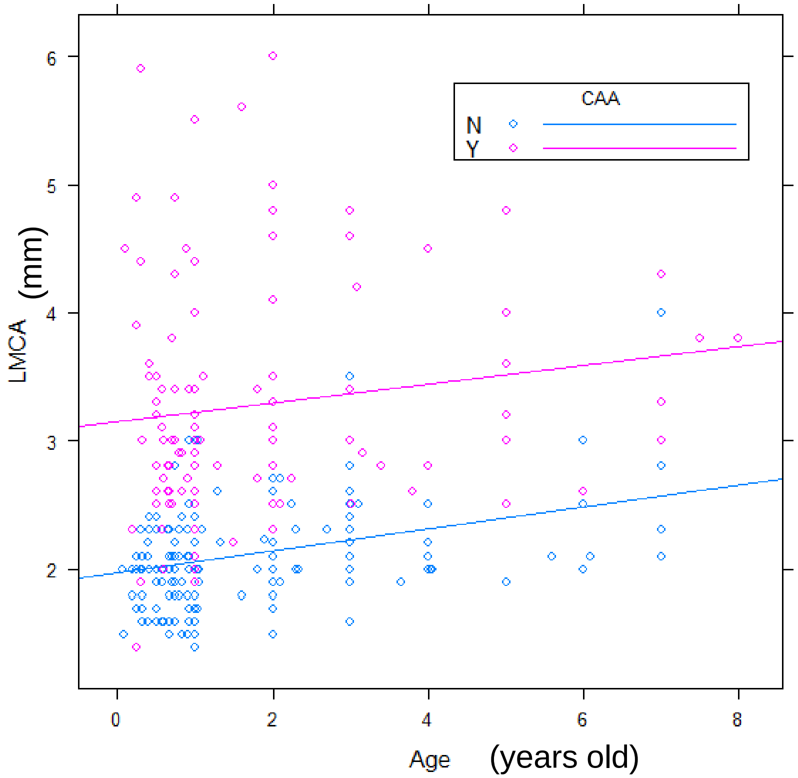

Figure S9

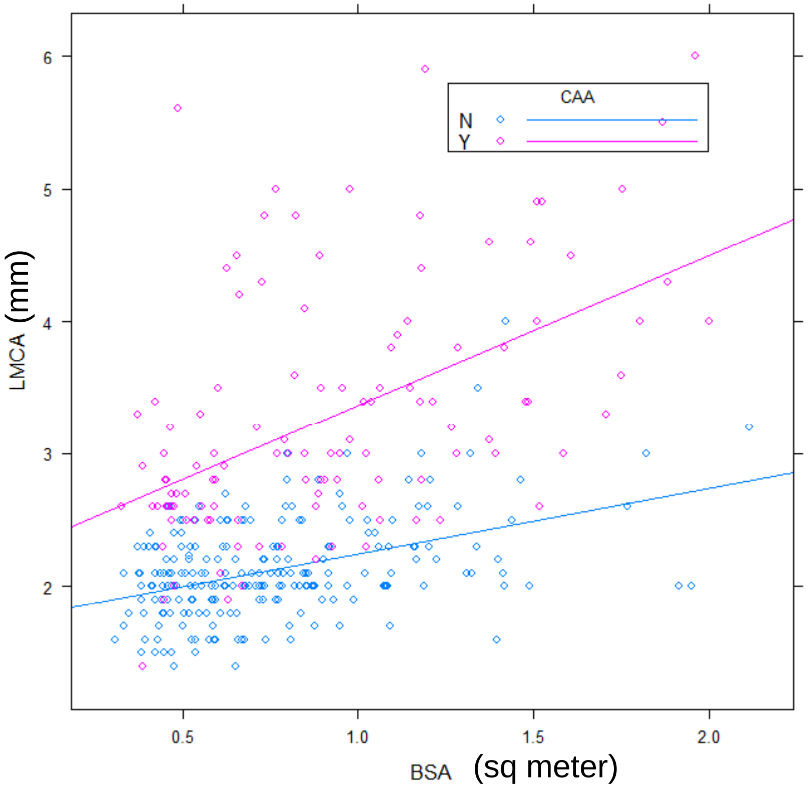

Figure S8. Left coronary artery (LCA) diameter verus age. KD with CAA group were with red line. KD without CAA group were with blue line.

Figure S9. Left coronary artery (LCA) diameter verus body surface area. KD with CAA group were with red line. KD without CAA group were with blue line.

Figure S10

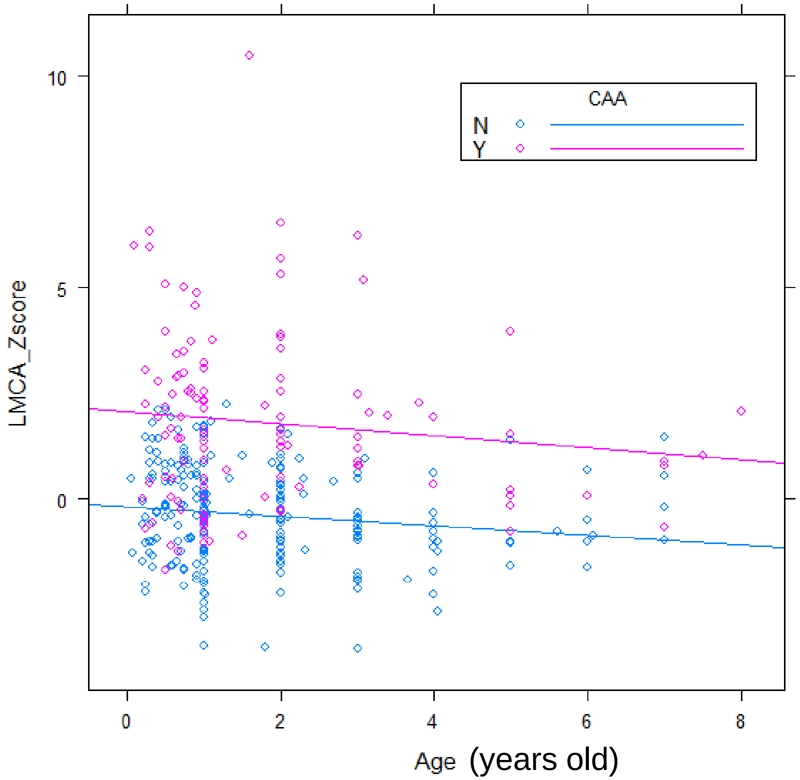

Figure S10. Left coronary artery (LCA) Z-score verus age. KD with CAA group were with red line. KD without CAA group were with blue line.

Figure S11

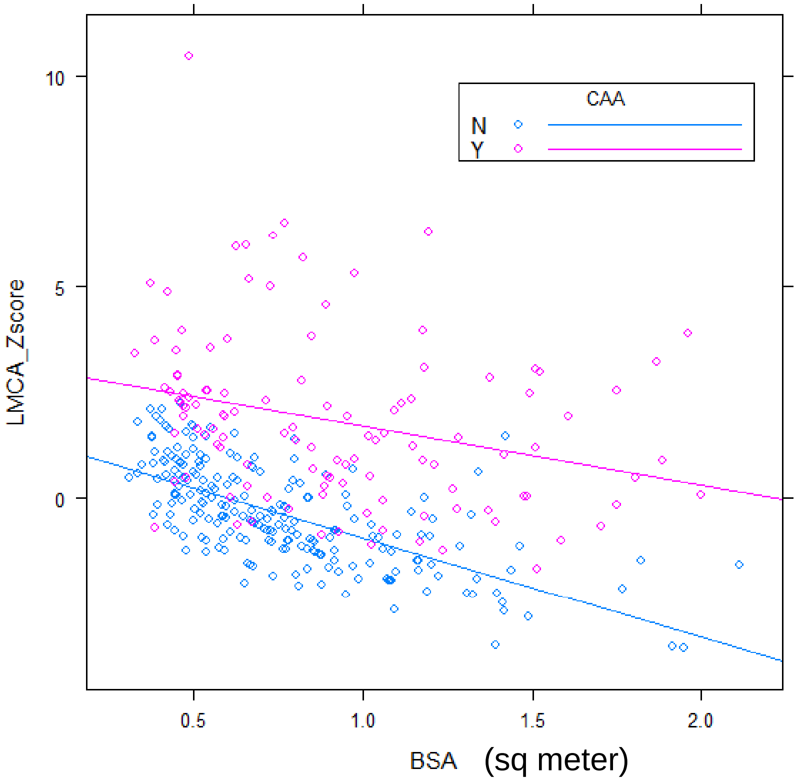

Figure S11. Left coronary artery (LCA) Z-score verus body surface area. KD with CAA group were with red line. KD without CAA group were with blue line.
